# Milder degenerative effects of Carfilzomib *vs*. Bortezomib in the *Drosophila* model: a link to clinical adverse events

**DOI:** 10.1038/s41598-017-17596-4

**Published:** 2017-12-19

**Authors:** Eleni N. Tsakiri, Evangelos Terpos, Eleni-Dimitra Papanagnou, Efstathios Kastritis, Vincent Brieudes, Maria Halabalaki, Tina Bagratuni, Bogdan I. Florea, Herman S. Overkleeft, Luca Scorrano, Alexios-Leandros Skaltsounis, Meletios A. Dimopoulos, Ioannis P. Trougakos

**Affiliations:** 1 Department of Cell Biology and Biophysics, Faculty of Biology, National and Kapodistrian University of Athens, 15784 Athens, Greece; 2 Department of Clinical Therapeutics, School of Medicine, National and Kapodistrian University of Athens, 11528 Athens, Greece; 3 Department of Pharmacognosy and Natural Products Chemistry, Faculty of Pharmacy, National and Kapodistrian University of Athens, 15771 Athens, Greece; 4 Leiden Institute of Chemistry and Netherlands Proteomics Centre, Gorlaeus Laboratories, Einsteinweg 55, 2333 CC Leiden, The Netherlands; 5 Dulbecco-Telethon Institute, Venetian Institute of Molecular Medicine and Department of Biology, University of Padua, 35129 Padova, Italy

## Abstract

Proteasome inhibitors, e.g. Bortezomib (BTZ) and Carfilzomib (CFZ), have demonstrated clinical efficacy against haematological cancers. Interestingly, several adverse effects are less common, compared to BTZ, in patients treated with CFZ. As the molecular details of these observations remain not well understood we assayed the pathophysiological effects of CFZ *vs*. BTZ in the *Drosophila* experimental model. Mass Spectrometry analyses showed that neither CFZ nor BTZ are hydrolysed in flies’ tissues, while at doses inducing similar inhibition of the rate limiting for protein breakdown chymotrypsin-like (CT-L) proteasomal activity, CFZ treatment resulted in less intense increase of oxidative stress or activation of antioxidant and proteostatic modules. Also, despite comparable cardiotoxicity likely due to disrupted mitochondrial function, CFZ did not affect developmental processes, showed minimal neuromuscular defects and reduced to a lesser extent flies’ healthspan. Studies in flies, human cancer cell lines and blood cells isolated from Multiple Myeloma patients treated with CFZ or BTZ revealed, that the increased BTZ toxicity likely relates to partial co-inhibition of the caspase-like (C-L) proteasomal activity Supportively, co-treating flies with CFZ and a C-L selective proteasome inhibitor exacerbated CFZ-mediated toxicity. Our findings provide a reasonable explanation for the differential adverse effects of CFZ and BTZ in the clinic.

## Introduction

Organisms require efficient surveillance of proteome quality to prevent disruption of proteostasis (homeodynamics of the proteome). Proteostasis maintenance is achieved by the concerted action of a number of modules that constitute the proteostasis network (PN). PN is (among others) composed from the network of molecular chaperones, as well as from the autophagy lysosome- (ALP) and the ubiquitin proteasome- (UPP) degradation pathways^1,2^. ALP is mostly involved in the clearance of protein aggregates and damaged organelles^3^, while UPP degrades normal short-lived ubiquitinated proteins and non-repairable unfolded polypeptides^1,4^. The 26 S proteasome is composed from the 20S core particle (CP) and the 19 S regulatory particles (RP)^5^. Proteasome peptidase activities are localised in the 20S CP and specifically at the β1, β2 and β5 subunits that bear caspase- (C-L; LLE/β1), trypsin- (T-L; LRR/β2) and chymotrypsin- (CT-L; LLVY/β5) like proteolytic activity, respectively^6,7^. Proteome functionality also depends on the transcriptional activity of the NF-E2-related factor 2 (Nrf2) which upon increased oxidative or proteotoxic stress stimulates the expression of antioxidant enzymes and proteasomal subunits^8,9^.

Recent findings indicate that over-activation of the proteostatic modules (e.g. UPP) represents a hallmark of advanced tumours; and thus, their inhibition provides a strategy for the development of novel anti-tumour therapies^10^. In line with this notion, proteasome inhibitors, e.g. Bortezomib (BTZ) and Carfilzomib (CFZ) have demonstrated clinical efficacy in the treatment of multiple myeloma (MM) and mantle cell lymphoma and are under evaluation for the treatment of other malignancies^11,12^. CFZ is an irreversible tetrapeptide epoxyketone–based proteasome inhibitor (analogue of Epoxomicin) which has demonstrated a high degree of clinical activity in patients with relapsed and/or refractory MM^11^. On the other hand, BTZ is a slowly reversible boronated proteasome inhibitor also showing significant clinical efficacy against haematological malignancies^13,14^. Most proteasome inhibitors (including CFZ and BTZ) were designed to target the CT-L activity since this site seems to be the rate limiting for protein breakdown^15,16^. Nevertheless, at higher concentrations these inhibitors may also inhibit the C-L or the T-L sites, or both; notably, the biological roles of C-L and T-L sites and their potential as co-targets of antineoplastic agents are not well defined^17–19^. Furthermore, while the use of proteasome inhibitors in the clinic during therapeutic treatment of MM patients is linked with several adverse effects, e.g. peripheral neuropathy, fatigue, cardiac failure and/or renal failure^11,12^, these effects are more enhanced or even almost exclusively seen (e.g. peripheral neuropathy) after BTZ treatment^20–23^; yet, the molecular basis of these intriguing clinical observations is not well understood.

As we recently reported that administration of BTZ to young *Drosophila* flies caused disruption of proteostasis, reduced motor function (a phenotype that recapitulates the peripheral neuropathy seen in the clinic) and a marked reduction of flies’ lifespan^9^; we sought to comparatively assay the pathophysiological effects of CFZ and BTZ in the fly *in vivo* experimental model. *Drosophila* is well-suited to this line of investigation, due to its powerful genetics; its similarities in key metabolic and ageing pathways with humans^24^, the fact that it expresses proteasomes that structurally resemble those from mammals^25^, and also because it comprises a soma-germ line demarcation composed of both post-mitotic and mitotic cells. Furthermore, apart from significant similarities in organs that perform the equivalent functions of the mammalian heart, lung, kidney, gut, and reproductive tract, flies are characterised by a well-developed and complicated neural system^26^. Moreover, they live for few months and thus, drug screening on large cohorts can be completed in a reasonable time^27,28^.

Herein, we report that under conditions of comparable inhibition of the CT-L proteasome activity in flies’ tissues, CFZ treatment resulted (as compared to BTZ) in less intense activation of antioxidant and proteostatic modules. Also, despite comparable cardiotoxicity, CFZ did not affect developmental processes, reduced to a lesser extent flies’ longevity and showed minimal neuromuscular defects. These findings likely relate to increased specificity of CFZ (*vs*. BTZ) for the CT-L proteasome activity.

## Results

### Under conditions of comparable CT-L inhibition, the CFZ-mediated molecular responses are milder compared to those observed after BTZ administration

**Figure 1:**
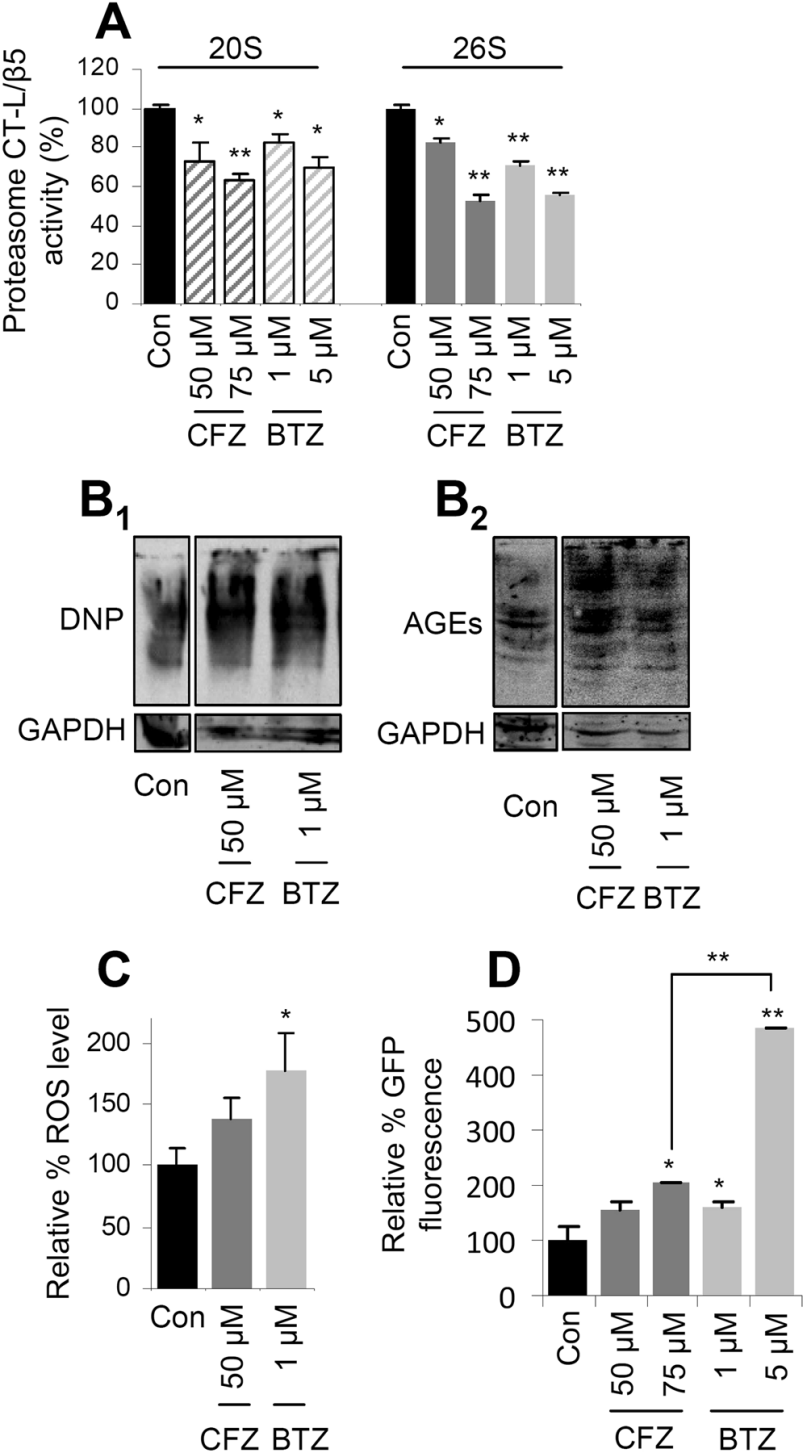
Under conditions of similar CT-L inhibition, CFZ induced milder, as compared to BTZ, antioxidant responses and less intense accumulation of ROS. (**A**) Relative (%) 20S and 26 S CT-L (LLVY/β5) activity in flies treated with the indicated doses of CFZ or BTZ for 7 days. (**B**) Representative immunoblot analyses of somatic tissues samples from young flies treated as in (**A**); protein samples were probed with antibodies against Dinitrophenol (DNP) or AGEs. (**C**) Relative (%) ROS levels in the somatic tissues of young flies exposed to shown doses of CFZ or BTZ for 18 days. (**D**) Relative (%) green fluorescent protein (GFP) levels in the somatic tissues of young transgenic gstD-ARE:GFP reporter flies that were treated with the indicated concentrations of CFZ or BTZ for 10 days. Control samples were set to 100%; GAPDH probing (**B**) was used as reference for total protein input. Comparisons were *vs*. control samples from flies grown in inhibitor-free culture medium. Bars, ±SD (n ≥ 2). *P < 0.05; **P < 0.01.

To comparatively assay the effects of CFZ and BTZ (Supplementary Fig. S1A) in flies’ tissues, we focused on the CT-L proteasome activity that reportedly is selectively targeted by these inhibitors^17,29,30^. As we had previously observed that BTZ was effective in partially suppressing the CT-L proteasome activity in the range of 1–5 μM^9^ we initially administered CFZ in flies at these concentrations; yet, we found no significant inhibitory effect on the CT-L activity. After screening a wide range of CFZ doses we observed that it induced an inhibitory effect on CT-L proteasome activity similar to that of 1 and 5 μM of BTZ at concentrations of 50 and 75 μM, respectively (Fig. 1A). We also found that CFZ addition in flies’ culture medium did not affect the rate of food consumption (Supplementary Fig. S1B) excluding thus the possibility of adverse effects due to caloric restriction.

We then sought to investigate the stability of BTZ and CFZ in flies’ culture medium and also to analyse whether drugs are hydrolysed and, if not, which is their concentration in flies’ tissues. Our high resolution UPLC-MS analyses showed that both drugs were stable in the culture medium (Supplementary Fig. S2A) and that in spite of oral administration, they bypass the gastric barrier and they are not hydrolysed, as they were found in flies’ tissues (Supplementary Fig. S2B) at concentrations ranging (depending on the input in the culture medium) from 9.1 to 33.8 nM for BTZ and from 61.8 to 75 nM for CFZ (Supplementary Fig. S2C).

**Figure 2:**
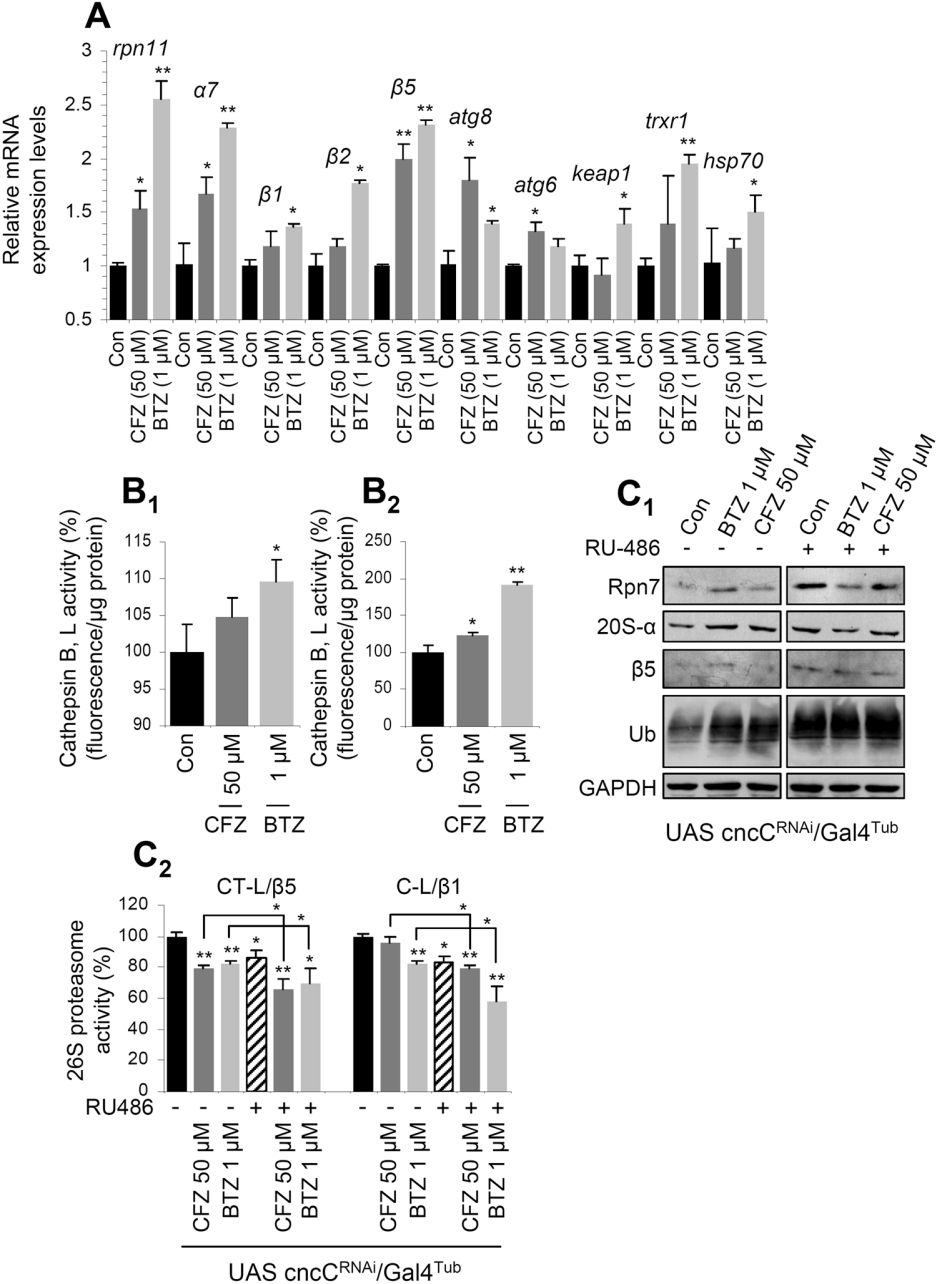
Treatment with CFZ promoted milder (as compared to BTZ) upregulation of antioxidant and proteostatic cellular modules. (**A**) Relative expression levels of the *rpn11*, *α7*, *β1*, *β2* and *β5* proteasome genes; *atg8* and *atg6* autophagic genes; *keap1* and *trxr1* antioxidant responses related genes and also of the molecular chaperone *hsp70* gene in the somatic tissues of young flies exposed to CFZ or BTZ for 24 days. (**B**) Relative (%) activities of lysosomal cathepsins B, L in flies’ somatic tissues after treatment with CFZ or BTZ for 9 (**B**_**1**_) or 24 (**B**_**2**_) days. (**C**) Representative immunoblots showing Rpn7, 20S-α, β5 and ubiquitin (Ub) expression levels (**C**_**1**_) and relative (%) CT-L (LLVY/β5) and C-L (LLE/β1) activities (**C**_**2**_) in somatic tissues of young transgenic flies after knocking down CncC/Nrf2; transgenic flies were fed (or not) with RU486 (to induce transgene expression) for 2 days and were then exposed (in the presence -or not- of RU486) to the indicated doses of CFZ or BTZ for 6 days. GAPDH probing (**C**_**1**_) and *rp49* gene (**A**) expression were used as reference for protein and RNA input, respectively. Shown comparisons refer to experimental *vs*. control samples from flies grown in inhibitor-free culture medium. Bars, ±SD (n ≥ 2). *P < 0.05; **P < 0.01.

By studying the molecular effects of CFZ in young flies’ tissues, we noted that (as BTZ) it induced the accumulation of carbonylated proteins (Fig. 1B_1_) and Advanced Glycation End-products (AGEs; Fig. 1B_2_). However, CFZ induced milder (compared to BTZ) induction of Reactive Oxygen Species (ROS) (Fig. 1C) and a significantly less intense activation of Antioxidant Response genomic Elements (AREs) in a reporter transgenic [(gstD1)-ARE: GFP/II] fly line (Fig. 1D). In support, gene expression analyses revealed a milder CFZ-mediated upregulation of proteasomal (*rpn11*, *α7*, *β1*, *β2*, *β5*), antioxidant (*keap1*, *trxr1*) and chaperone (*hsp70*) genes (Fig. 2A), and a less intense activation of lysosomal cathepsins (Fig. 2B). As in the case of BTZ treatment^9^, the CFZ-mediated responses on proteasome subunits upregulation were dependent on cap ‘n’ collar isoform-C (CncC; the Nrf2 ortholog in flies) transcriptional activity, since they were largely abolished after RNAi-mediated knock down (KD) of CncC/Nrf2 (Fig. 2C_1_). In support, the achieved levels of proteasome inhibition were enhanced under conditions of CncC/Nrf2 KD (Fig. 2C_2_). The CFZ-mediated proteasome subunits upregulation was (as for BTZ^9^) age-dependent, since despite effective suppression of the CT-L activity (Supplementary Fig. S3A_1_,B_1_), the proteasome subunits were not significantly induced in CFZ-treated middle aged (Supplementary Fig. S3A_2_) or aged flies’ tissues (Supplementary Fig. S3B_2_). Considering that the CncC/Nrf2 antioxidant pathway is deregulated during ageing^9,31^, we hypothesise that the abolishment of proteasome upregulation after pharmacological proteasome inhibition in aged flies is likely due to CncC/Nrf2 dysfunction.

Taken together these data indicate that under conditions of comparable CT-L inhibition the (age- and CncC/Nrf2-dependent) CFZ-mediated molecular responses are milder compared to those observed after BTZ administration.

### CFZ shows superiority over BTZ on toxic effects

**Figure 3:**
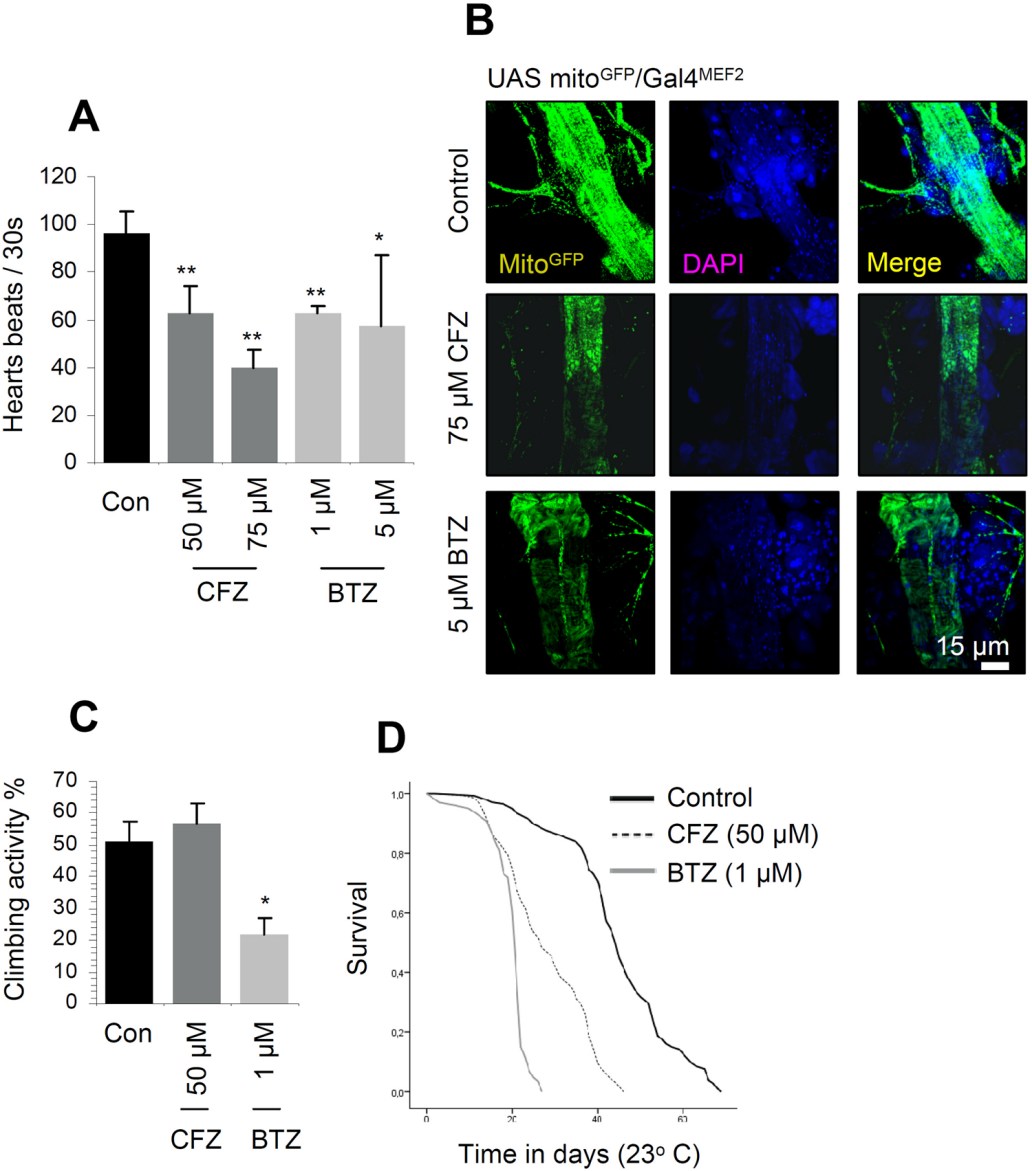
Both CFZ and BTZ caused cardiac toxicity; yet, CFZ showed minimal neuromuscular defects and reduced to a lesser extent flies’ longevity. (**A**) Heart beats (mean values) normalised to a 30 sec period; increasing doses of CFZ or BTZ (exposure for 14 days) led to reduced rate of heart beats and arrhythmia (see also, Supplementary Videos S1–S3). (**B**) CLSM visualization of isolated heart tubes from young UAS mito^GFP^/Gal4^MEF2^ reporter flies that were exposed for 14 days to the shown concentrations of CFZ or BTZ; nuclei were counterstained with DAPI. (**C**) Climbing (%) (locomotion performance) activity of middle aged flies that were treated with the indicated concentrations of CFZ or BTZ for 24 days. (**D**) Longevity curves of flies exposed to the indicated concentrations of CFZ or BTZ; comparative statistics of the longevity assays are reported in Supplementary Table S1. Comparisons were *vs*. control samples from flies grown in inhibitor-free culture medium. Bars, ±SD (n ≥ 2). *P < 0.05; **P < 0.01.

We then comparatively analysed the pathophysiological effects of CFZ *vs*. BTZ. Since a severe (yet, less studied) adverse effect of therapeutic proteasome inhibition relates to cardiotoxicity^21,23^ we initially focused on analysing the effects on fly hearts. As shown in Fig. 3A prolonged treatment with either CFZ or BTZ caused significant cardiotoxicity, including a reduction of the heart beats (Fig. 3A), arrhythmia and (at least in CFZ treated flies) in periods of asystole where heart beating temporarily ceased (Supplementary Videos S1–S3). As cardiomyocytes are heavily dependent on proper mitochondria function^32,33^, we investigated the effect of CFZ and BTZ treatment on mitochondria of heart tubes isolated from transgenic flies bearing a mito^GFP^ reporter expressed in muscle cells (Gal4^MEF2^ driver). As shown in Fig. 3B, CLSM studies revealed that both proteasome inhibitors caused a significant reduction in mitochondria number and also affected the overall morphology of heart tubes indicating that proper proteasome functionality is central to maintenance of mitostasis. In line with these findings we observed that treatment with BTZ severely affected mitochondrial respiration in adult flies (Supplementary Fig. S4A) and *in vivo* O_2_ consumption in larvae (Supplementary Fig. S4B). These effects were further confirmed by Electron Microscopy studies in adult flies muscle tissues where we observed that treatment with either BTZ or CFZ resulted in severe disruption of mitochondria structure (Supplementary Fig. S4C; upper panels) and reduced mean length of mitochondria (Supplementary Fig. S4C; lower panels). These features indicate that the reduced mitochondrial numbers in flies’ heart tubes after treatment with CFZ or BTZ are likely due to enhanced mitochondrial fission and increased rates of mitophagy.

As BTZ had shown severe toxic effects when administered during early developmental stages of flies^9^, we also investigated the impact of CFZ administration during flies’ development and found that it exerted no major toxicity. Specifically, we observed normal eclosion after the administration of either 50 or 75 μM of CFZ (Supplementary Fig. S5A). Also, flies that had completed development in the presence of CFZ and were then grown in inhibitor-free medium showed no reduced longevity as compared to controls that concluded development in physiological culture medium (Supplementary Fig. S5B; see also, Supplementary Table S1).

Another major adverse effect of therapeutic BTZ in MM patients is the development of peripheral neuropathy^11^. Indeed, our previous studies^9^ had shown that BTZ administration in adult flies resulted in severe decrease of climbing activity, indicating reduced neuromuscular performance that recapitulates peripheral neuropathy of BTZ treatment in the clinic; also, treatment of flies with BTZ resulted in significant dose-dependent decrease in flies’ longevity^9^. Interestingly, our comparative studies showed that exposure of flies to 50 μM CFZ exerted no significant effect on their climbing activity (neuromuscular performance) (Fig. 3C) and caused a significantly less intense (compared to 1 μM BTZ) reduction in flies’ healthspan and longevity (Fig. 3D).

Overall, under conditions of comparable CT-L inhibition, and despite similar cardiotoxicity likely due to mitochondrial dysfunction, CFZ administration in flies results in less severe (compared to BTZ) toxic effects on the neuromuscular system, as well as in reduced mortality rates.

### The milder degenerative effects of CFZ likely relate to its increased selectivity (compared to BTZ) for the CT-L proteasomal activity

Reportedly, BTZ can also inhibit the C-L or the T-L proteasome sites^9,17–19^. Thus, we investigated the CFZ effect on these proteasomal peptidases. At *in vitro* assays in *Drosophila* tissues cell lysate containing intact proteasomes, CFZ showed increased selectivity (although it also modestly inhibited the C-L and T-L activities), as compared to BTZ, against the CT-L activity (Supplementary Fig. S6). In these series of experiments, we also found that Epoxomicin (a CFZ analogue) was also selective against the CT-L activity (Supplementary Fig. S6).

**Figure 4:**
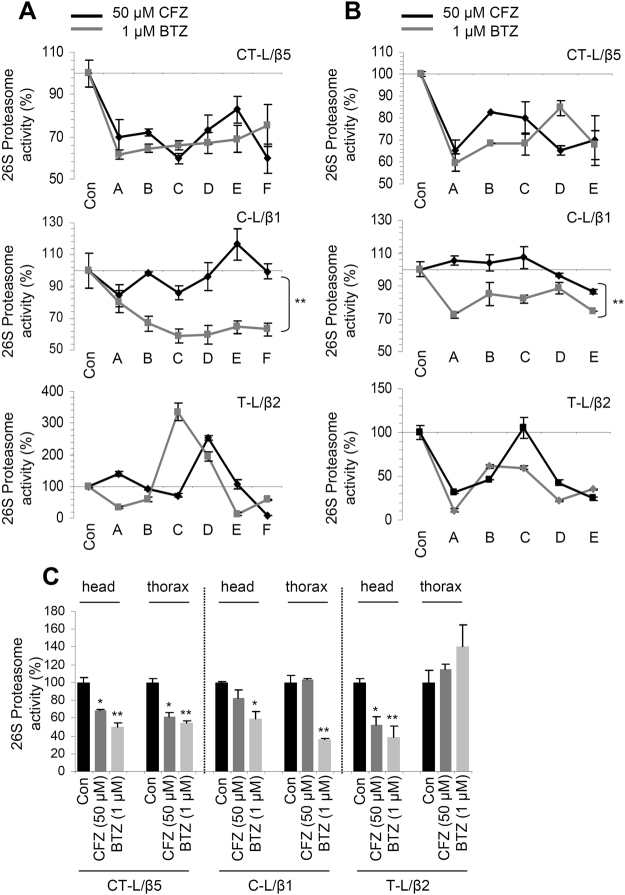
Comparative short and long term effects of CFZ and BTZ to proteasome peptidases activity in flies’ somatic tissues. (**A**,**B**) Relative (%) CT-L (LLVY/β5), C-L (LLE/β1) and T-L (LRR/β2) proteasome activities, in flies’ somatic tissues following treatment with the indicated concentrations of CFZ or BTZ according to the protocol described in Supplementary Fig. S7A,B, respectively. In (A), flies were continuously exposed to the shown inhibitors and were transferred to fresh culture medium (containing –or not– the drug) every 4 days. A-F denotes time points (see also arrows in Supplementary Fig. S7A) of proteasome activities measurement as follows: *A*: Exposure for 1 day to the PI; *B*: exposure for 4 days [in the culture medium of (A)] to the PI; *C*: exposure for 5 days to the PI (1 day in fresh culture medium containing the PI); *D*: exposure for 8 days [4 days in the culture medium of (C)] to the PI; *E*: exposure for 9 days to the PI (1 day in fresh culture medium containing the PI); *F*: exposed for 12 days [4 days in the culture medium of (E)] to the PI. In (B), flies were exposed for a period of 72 hrs to the shown drugs and were then transferred to inhibitor-free culture medium. A-E indicates time points (see also arrows in Supplementary Fig. S7B) of proteasome activities measurement as follows: *A*: Exposure for 3 days to the shown PI; *B*: flies from (A) exposed for 1 day to inhibitor-free culture medium; *C*: flies from (A) exposed for 2 days to inhibitor-free culture medium; *D*: flies from (A) exposed for 3 days to inhibitor-free culture medium; and *E*: flies from (A) exposed for 4 days to inhibitor-free culture medium. (**C**) Relative (%) CT-L (LLVY/β5), C-L (LLE/β1) and T-L (LRR/β2) proteasome activities in dissected head and thorax preparations; CFZ or BTZ were administered to young flies at the shown doses for 13 days. PI, proteasome inhibitor; Con, indicates inhibitor-free culture medium. Bars, ±SD (n ≥ 2). *P < 0.05; **P < 0.01.

To investigate the *in vivo* effects of CFZ (*vs*. BTZ) on the C-L and T-L proteasomal activities we exposed flies to proteasome inhibitors either continuously for 12 days, or for three days followed by prolonged culturing in inhibitor-free medium; proteasome activities were then measured at several time points as indicated in Supplementary Fig. S7. We found that under these experimental conditions and doses, CFZ was increasingly selective against the CT-L activity, while BTZ also affected the C-L proteasome activity; notably, for both inhibitors, T-L activity seemed to fluctuate in a CT-L and C-L independent pattern (Fig. 4A,B). Furthermore, both inhibitors were rather stable in young flies’ tissues, as at day four post-exposure to CFZ or BTZ and culturing in inhibitor-free medium, proteasome activities were still suppressed (Fig. 4B). These findings were also verified after assaying proteasome activities in dissected head (mostly neuronal tissue with minor contributions from fat and muscles) and thorax (mainly muscle tissue and the tracheal system) preparations of CFZ or BTZ treated flies (Fig. 4C; note that CFZ and BTZ also inhibited T-L activity in head tissues). These data are additive to our UPLC-MS findings (Supplementary Fig. S2) as they indicate that orally administered proteasome inhibitors are similarly distributed in the various body parts of young flies.

Given the fact that RNAi-mediated KD of either the β5 (CT-L activity) or the β1 (C-L activity) proteasomal subunits was developmentally lethal^9^, we used the C-L selective inhibitor, NC001 (Supplementary Fig. S8; upper panel) in order to study the effects of selective C-L inhibition^34^ in adult flies pathophysiology. We observed that addition of NC001 in fly tissues lysate containing intact proteasomes resulted in selective inhibition (at least at the lower concentrations of 0.05–0.1 μM) of the C-L activity (Supplementary Fig. S8; lower panel). NC001 was also found to demonstrate (at lower concentrations) increased selectivity against the C-L activity at *in vivo* (supplementation in flies’ culture medium) assays (Supplementary Fig. S9A). In line with the notion that CT-L is the rate limiting proteasome activity for protein breakdown^15,16^, treatment of flies with NC001 did not significantly affect (at 2–4 μM) development (not shown) or cellular ROS levels (Supplementary Fig. S9B) and only marginally upregulated proteasomal subunits expression (Supplementary Fig. S9C). NC001 increased the mortality rate of flies, albeit less intensively as compared to either BTZ or CFZ (Supplementary Fig. S9D). However, co-treatment of flies with both CFZ and NC001 increased the mortality rate of CFZ (Supplementary Fig. S9E), further supporting the notion that the decreased toxicity of CFZ relates to its increased selectivity for the CT-L proteasome activity.

**Figure 5:**
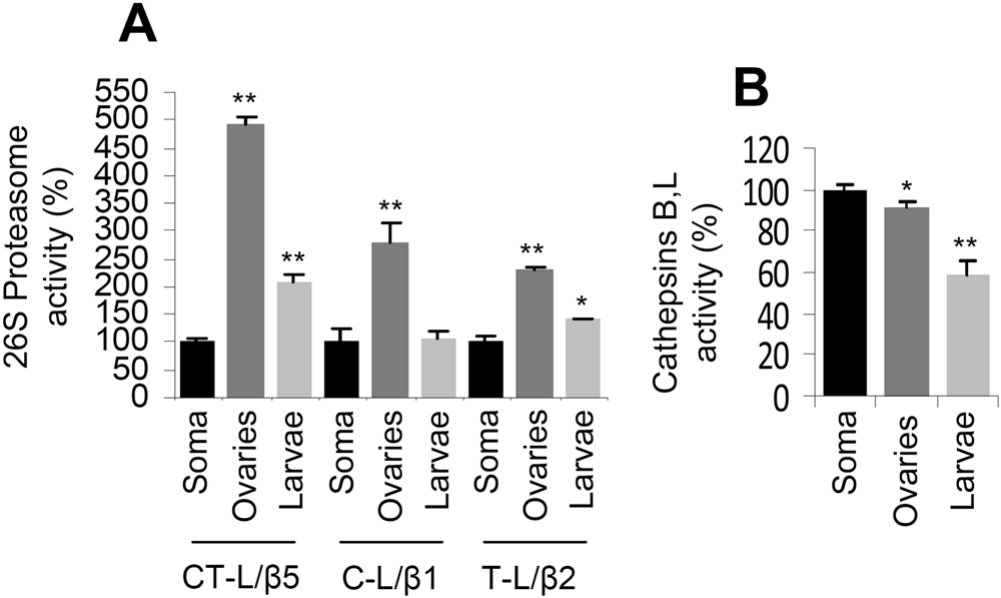
Proteasome and lysosomal cathepsins enzymatic activities in adult flies’ tissues or developmental stages (ovaries and larvae) enriched in mitotic (ovaries and larvae) or post-mitotic (adult soma) cell lineages. (**A**) Relative (%) activity of the CT-L (LLVY/β5), C-L (LLE/β1) and T-L (LRR/β2) proteasome peptidases in adult somatic tissues, ovaries and larvae. (**B**) Relative (%) lysosomal cathepsins B, L activities in adult somatic tissues, ovaries and larvae. In all cases comparisons were *vs*. values (set to 100%) in adult somatic tissues. Bars, ±SD (n ≥ 2). *P < 0.05; **P < 0.01.

To get more insights into the distinct pattern of toxicity between CFZ and BTZ, we also assayed the basal levels of proteasome and lysosomal cathepsins activities in fly tissues or developmental stages enriched in either mitotic (ovaries, larvae) or post-mitotic (adult somatic tissues) cells. Interestingly, we noted that both ovaries and larvae express significantly higher basal proteasome activities as compared to adult somatic tissues (Fig. 5A). On the contrary, adult somatic tissues are characterised by elevated (*vs*. ovaries or larvae) lysosomal cathepsin activities (Fig. 5B). Thus, it is anticipated that mitotic cells (including highly proliferating tumour cells) would be increasingly sensitive to proteasome inhibition compared to post-mitotic somatic cells. On the other hand, post-mitotic tissues (e.g. neuronal cells) would be likely increasingly sensitive to inhibitors suppressing both CT-L and C-L proteasomal activities and of course to ALP inhibition.

**Figure 6:**
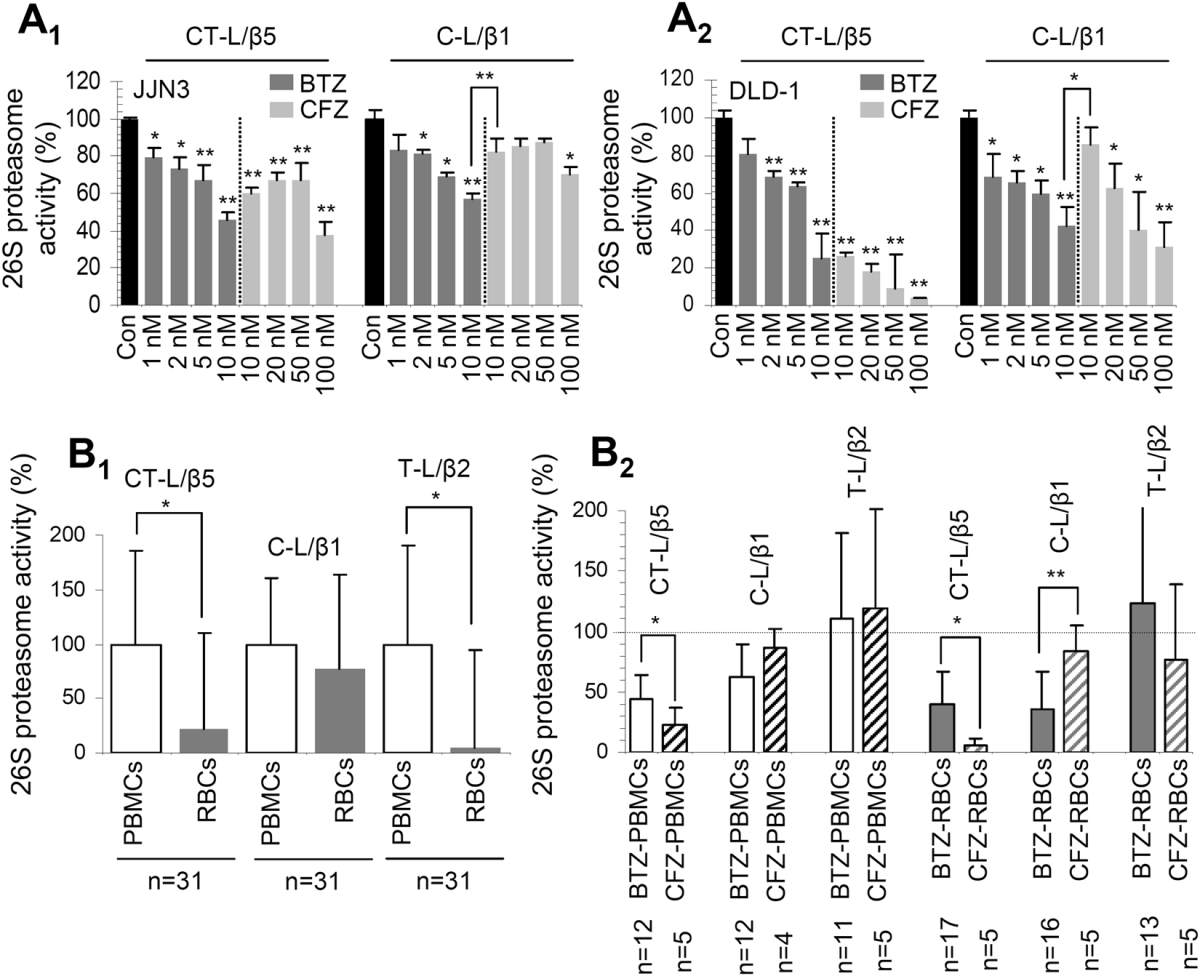
CFZ is increasingly selective (compared to BTZ) for the CT-L proteasome activity in human cancer cell lines or in PBMCs/RBCs (PBMCs express higher proteasomal activities *vs*. RBCs) isolated from patients treated with therapeutic CFZ or BTZ. (**A**) Relative CT-L and C-L proteasomal activities in JJN3 (**A**_**1**_) and DLD-1 (**A**_**2**_) tumour cell lines treated with the shown concentrations of BTZ or CFZ. Shown comparisons were *vs*. basal proteasomal activities in non-treated cells. (**B**_**1**_) Relative (%) basal activity of the CT-L (LLVY/β5), C-L (LLE/β1) and T-L (LRR/β2) proteasome peptidases in isolated PBMCs and RBCs. (**B**_**2**_) Relative (%) CT-L, C-L and T-L proteasome activities in PBMCs and RBCs isolated from MM patients after receiving therapeutic doses of BTZ or CFZ for 24 hrs. In (B_1_) normalization was *vs*. values (set to 100%) in PBMCs; in (B_2_) normalization was *vs*. values before treatment. Samples in (**B**) were from both male and female donors. Bars, ±SD. *P < 0.05; **P < 0.01.

We sought to further verify our findings in the fly model, in human cancer cell lines and in peripheral blood mononuclear cells (PBMCs) and red blood cells (RBCs) isolated from MM patients prior and after therapeutic treatment with CFZ or BTZ. In accordance to our observations in flies, treatment of the MM JJN3 (Fig. 6A_1_) or the colon adenocarcinoma DLD-1 (Fig. 6A_2_) cells with BTZ or CFZ revealed an increased specificity of CFZ (compared to BTZ) against the CT-L proteasomal activity. Furthermore, we noted that PBMCs express higher basal proteasome activities as compared to RBCs (Fig. 6B_1_), and CFZ was increasingly selective (compared to BTZ) against CT-L in both PBMCs and RBCs (Fig. 6B_2_).

Taken together these data suggest that the CFZ superiority over BTZ on toxic effects likely relates to its increased selectivity for the CT-L proteasome activity.

## Discussion

UPP is a key regulator of cellular proteostasis and thus a major regulatory hub for cellular survival in both physiological and cancer cells. Thus, it is not surprising that the usage of proteasome inhibitors has revolutionised cancer therapy over the last two decades^1,35^. BTZ and CFZ have demonstrated clinical efficacy in the treatment of haematological cancers and are under evaluation for other malignancies^36,37^. Nonetheless, their therapeutic profile and demonstrated adverse effects in the clinic are dissimilar^11^. Herein, we have comparatively analysed the CFZ and BTZ effects in *Drosophila* flies and found that after oral administration they are not hydrolysed in flies’ tissues. Also, as in the clinic, under conditions of comparable CT-L inhibition, CFZ was less toxic as compared to BTZ. These findings further validate the fly model as an *in vivo* experimental platform for screening the efficacy and/or safety of proteasome inhibitors and/or other drugs^9,27^. This notion is further supported by the finding that the ratio of the different doses used to achieve similar rates of CT-L inhibition in *Drosophila* somatic tissues (i.e. 1 μM BTZ *vs*. 50 μM CFZ in culture medium) is reminiscent to the therapeutic doses used in the clinic (i.e. ~1.3 mg/m^2^ BTZ *vs*. ~25–50 mg/m^2^ CFZ).

Despite differences in the intensity of molecular responses, both CFZ and BTZ mobilised antioxidant and proteostatic modules in a CncC/Nrf2 and, more importantly, age-dependent manner. In support, CFZ and BTZ have been previously implicated in ROS production and induction of Hsp70^38,39^. Also, these inhibitors likely activate prosurvival autophagy as can be assumed from upregulation of autophagic genes and of lysosomal cathepsins activity in flies’ tissues. Similarly, it was shown previously in mammalian cells^40^ that CFZ induced autophagy via induction of the Unfolded Protein Response. These findings broaden the potential means for increasing the therapeutic window against malignancies, e.g. by also using inhibitors of ALP or of the cytoprotective CncC/Nrf2 pathway. Consistently, a recent study revealed that a combination therapy with CFZ plus Chloroquine (an ALP inhibitor) was highly effective in the treatment of MM in a mouse xenograft model^41^. Nevertheless, these therapeutic approaches should be applied with caution as according to our findings post mitotic cells appear to be increasingly dependent on ALP functionality^42^ and so there is an increased risk of serious toxic effects. Furthermore, our data raise the significant issues of, *a*. directly measuring (or even better adjusting) the levels of achieved reduction in targeted proteasomal activities after administration of therapeutic inhibitors and not just delivering drugs in mg/m^2^ doses and; *b*. considering the parameter of age during therapy, as it is anticipated that, due to age-dependent reduction in genomic responses, the toxicity of the inhibitors would likely increase in aged patients.

Our finding that CFZ did not significantly affect the climbing activity of flies indicates no significant pathologies in *Drosophila* muscle, peripheral neurons or central nervous system. Moreover, CFZ did not exert significant effects during developmental stages, while, as compared to BTZ, its administration in adult flies promoted the increase of their mortality rate to a lesser extent. In support, administration of therapeutic doses of CFZ in MM patients is not associated with the development or significant worsening (if it follows BTZ treatment) of peripheral neuropathy^14,22,43^. Also, according to the ENDEAVOR study the number of patients who had grade 2 or higher peripheral neuropathy (grouped term) was significantly higher in the BTZ group than in the CFZ group^44^. Interestingly, according to our findings both inhibitors caused cardiotoxicity in the fly model. This effect was manifested by bradycardia and/or arrhythmia and likely relates to reduced mitochondria number, as well as well as to significant disruption of mitochondrial structure and respiration machineries functionality. In the clinic, CFZ has been related to a low but reproducible signal of cardiotoxicity, associated with reduction of ejection fraction, which in most cases is reversible. Also, few studies have shown that CFZ and BTZ correlate with increased heart cytotoxicity and failure, respectively^20,21,23,45^. Moreover, in a primary neonatal rat myocytes model, exposure to BTZ or CFZ resulted in significant myocytes damage and induced apoptosis. Reportedly, the increased sensitivity of myocytes to proteasome inhibitors was likely due to inhibition of the proteasomal-dependent sarcomeric protein turnover^46^. Cardiomyocytes are constantly bearing a hard mechanical work and increased metabolic rate, and are thus increasingly depended on functional mitochondria and UPP for their homeodynamic maintenance^47^. Therefore, it is not surprising that proteasome inhibition can lead to heart remodelling, dysfunction or even failure^48,49^. Nonetheless, further studies are needed in order to clarify in detail the role of UPP (and its therapeutic inhibitors) in heart function.

Our data indicate that the milder toxicity of CFZ in the fly model is likely due to selective inhibition of the CT-L proteasome activity; this finding was also verified in human cancer cell lines exposed to BTZ or CFZ, and in RBCs or PBMCs isolated from MM patients treated with BTZ or CFZ. In support, a C-L selective inhibitor (NC001) increased the CFZ mortality rate in the fly model. Therefore, considering the CFZ therapeutic effects, it seems that haematological tumours are uniquely sensitive to CT-L inhibition. In agreement with this notion, CFZ at a dose that selectively inhibits both the CT-L active sites of β5 and LMP7 induced an anti-tumour response in MM, non-Hodgkin lymphoma (NHL) and leukaemia cells with minimal cytotoxic effects in non-transformed cells^50^. Nevertheless, although the CT-L site seems to be rate limiting for protein breakdown^15,51^, inhibiting CT-L alone is rarely sufficient to block protein degradation^19,52^. Thus, co-inhibition of either the C-L or T-L sites is usually required to effectively inhibit protein breakdown^19,52^ and to increase the sensitivity of other types of cancer cells (e.g. solid tumours) to inhibitors of the CT-L site, including BTZ and CFZ^53,54^; it is nonetheless anticipated that this approach will significantly enhance the toxic effects of the used inhibitors.

Interestingly, we found that mitotic cells express higher (as compared to post-mitotic cell lineages) proteasome activities, whereas post-mitotic cells are characterised by increased lysosomal cathepsins activity. It is thus logical to assume that highly proliferating tumour cells, would be addicted to high expression levels and activities of UPP^10,55–57^ (and thus differentially sensitive to its inhibition) due to enhanced protein synthesis and thus increased needs for protein synthesis quality control. On the other hand, post-mitotic tissues likely mostly depend on ALP for proteostasis maintenance and clearance of proteome damage^42^. This notion suggests that the adverse effects in post-mitotic tissues, e.g. nervous system, would be enhanced during therapeutic approaches which co-inhibit UPP and ALP in order to maximize anti-tumour therapy.

Taken together our data suggest that (Supplementary Fig. S10), compared to BTZ, administration of therapeutic CFZ exerts milder degenerative effects (except cardiac toxicity) in flies’ tissues likely due to increased selectivity for the CT-L proteasomal activity. These findings provide a reasonable explanation for the differential adverse effects of CFZ and BTZ in the clinic.

## Methods

### Proteasome inhibitors

BTZ (Velcade^®^) was obtained commercially and CFZ was from Onyx Pharmaceuticals, Inc. (an Amgen subsidiary). The inhibitors were diluted in ddH_2_O; aliquoted and stored at −20 °C. Daily used aliquots were stored at 4 °C. For the *in vivo* assays inhibitors were added in the fly culture medium.

### Fly Stocks

The *Drosophila* strains used in this study were the wild-type Oregon R and w^1118^ strains. The AREs (Antioxidant Response Elements)-GFP transgenic reporter line gstD-ARE:GFP/II (ARE-containing the enhancer of the *gstD1* gene) and the UAS cncC^RNAi^ (overexpress an inverted repeat corresponding to the *cncC/nrf2* gene^58^) line, along with the Tubulin GeneSwitch Gal4 (tubGSGal4) driver were a gift from Prof. D. Bohmann (University of Rochester, NY, USA). The tubulin-GeneSwitch-Gal4 (tubGSGal4) flies express RU486-regulated Gal4 under the control of the tubulin enhancer; thus, the conditional driver (tubGSGal4) is ubiquitously activated upon dietary administration of RU486 (320 μM). The Gal4^MEF2^ muscle-specific driver and the Mito^GFP^ reporter lines were a gift from Prof. A. Daga (University of Padua, Padova, Italy). As gonads display distinct regulation of proteostatic mechanisms and ageing rates compared to adult somatic tissues^59^, in all shown experiments (unless otherwise indicated) referring to adult flies only microdissected somatic tissues (head and thorax; equal numbers from mated male and female flies) were analysed.

### Flies culture, exposure to compounds, locomotion and longevity assays

Flies stocks were maintained at 23 °C, 60% relative humidity on a 12-h light: 12-h dark cycle and were fed standard medium (unless otherwise indicated)^60^. All used compounds, e.g. BTZ, CFZ or RU486 (Sigma-Aldrich, M8046) were added in flies’ culture medium. The duration of flies’ exposure to compounds and used doses are indicated in Figure legends.

Locomotion (climbing) and longevity assays were done as described previously^9,59,61^. For survival curves and statistical analyses the Kaplan-Meier procedure and log-Rank (Mantel-Cox) test were used; significance was accepted at *P* < 0.05. Statistical analyses of the lifespan experiments are presented in Supplementary Table S1.

### Cell lines and culture conditions

The human myeloma JJN3 cell line was a gift from Prof. C. Mitsiades (Dana-Farber Cancer Institute, Harvard Medical School, USA) and the colon adenocarcinoma DLD-1 cell line was obtained from the American Tissue Culture Collection (ATCC^®^). JJN3 and DLD-1 cells were maintained in RPMI-1640 medium containing 10% (v/v) foetal bovine serum and 2 mM glutamine. Cells were cultured in a humidified incubator at 5% CO_2_ and 37 °C. DLD-1 cell line was subcultured using a trypsin/EDTA solution, while a saturated JJN3 (grown in suspension) cell culture was collected after centrifugation and splitted 1:3 to 1:6 every 2-3 days^62^.

### PBMCs and RBCs isolation

PBMCs and RBCs were collected by using Biocoll [density 1.077 g/ml (Biochrom)] from whole blood isolated from MM patients upon diagnosis or 24 hrs post-CFZ or BTZ administration. Freshly collected heparinised blood was transferred into a 15 mL tube and was then diluted (1:1 dilution) with phosphate-buffered saline (PBS). The diluted whole blood was then carefully layered over the separation medium (1/2 x the volume of the sample); the two phases were kept separated before the centrifugation. Tubes were then centrifuged at 400 xg for 30 min at 20 °C (acceleration 9, braking rate 0). Both cell types were collected; washed with PBS and were then processed for proteasome peptidases activity measurement.

**Full Methods**, description of **Statistical analyses** and any associated **References** are available in Supplemental Methods.

### Ethics approval and consent to participate

Informed consent was obtained from all individual participants that were included in the study, which was conducted in accordance with the ethical standards of the institutional and/or national research committee and with the 1964 Helsinki Declaration and its later amendments or comparable ethical standards. An approval has been obtained from the Institutional Review Board/Scientific committee of Alexandra Hospital (Athens, Greece) for the collection of blood samples.

### Data availability statement

The datasets generated during and/or analysed during the current study are available from the corresponding authors on reasonable request.

## Electronic supplementary material

Supplementary Information

Suppl Video S1

Suppl Video S2

Suppl Video S3
