# Supplementary material for: Milder degenerative effects of Carfilzomib vs. Bortezomib in the Drosophila model: a link to clinical adverse events
Source: Sci Rep. 2017 Dec 19;7:17802. doi: 10.1038/s41598-017-17596-4 (PMC5736585; doi:10.1038/s41598-017-17596-4)
Supplement: Supplementary file 1 — Supplementary Information [file 41598_2017_17596_MOESM1_ESM.pdf]

Milder degenerative effects of Carfilzomib *vs.* Bortezomib in the *Drosophila* model: a link to clinical adverse events

Eleni N. Tsakiri, Evangelos Terpos, Eleni-Dimitra Papanagnou, Efstathios Kastritis, Vincent Brieudes, Maria Halabalaki, Tina Bagratuni, Bogdan I. Florea, Herman S. Overkleeft, Luca Scorrano, Alexios-Leandros Skaltsounis, Meletios A. Dimopoulos and Ioannis P. Trougakos

SUPPLEMENTARY INFORMATION

Supplementary Figure Legends

Suppl. Figure S1. Addition of CFZ in flies' culture medium did not affect the rate of food consumption. (A) Chemical structure of Bortezomib (BTZ) (A_1) and Carfilzomib (CFZ) (A_2). (B) Relative abdomen redness of flies (n=10 per assay) fed (or not) with the indicated doses of CFZ. Bars, ± SD (n = 3).

Suppl. Figure S2. CFZ and BTZ are stable in flies' culture medium and are not metabolised in treated flies' somatic tissues. (A). LC-ESI-MS chromatograms derived from the analysis of flies' culture medium samples. *Up*: superimposed chromatograms of control culture medium (black line), CFZ_50 μM (medium containing 50 μM CFZ) (red line) and CFZ_250 μM (medium spiked with 250 μM CFZ) (green line). *Down*: superimposed chromatograms of control medium (black line), BTZ_1 μM (medium containing 1 μM BTZ) (red line) and BTZ_5 μM (medium spiked with 5 μM BTZ) (green line). The MS spectra of CFZ and BTZ are also presented and the monitored ion transitions are given (CFZ, transition 720.5>289 and 720.5>261 and BTZ, transition 383.2>364.9 and 383.2>321.9). CFZ or BTZ are absent in control samples while they are clearly detected in culture media containing the respective analytes. (B) (*left*) ESI(-) chromatograms of CFZ (transition 720.5>289). A: Control flies' tissues preparations; B: Control flies' tissues plus spiked-in CFZ standard at 10 ng/mL; C: Samples from flies' tissues after treatment with 50 μM CFZ; D: Samples from flies' tissues after treatment with 75 μM CFZ. (*right*) ESI (+) chromatograms of BTZ (transition 383.2>364.9). A: Control flies' tissues preparations; B: Control flies' tissues plus spiked-in BTZ standard at 10 ng/mL; C: Samples from flies' tissues after treatment with 1 μM BTZ; D: Samples from flies' tissues after treatment with 5 μM BTZ. (C). Averaged concentrations (ng/mL; shown also in nM) of CFZ or BTZ in flies' tissues; samples were obtained from 50 flies each, exposed (or not) for 5 days to shown concentrations of CFZ or BTZ through diet. Bars, ± SD (n = 3).

Suppl. Figure S3. Upregulation of proteasome subunits after treating flies with proteasome inhibitors is age-dependent. (A) Relative (%) CT-L (LLVY/β5) activity (A_1) and immunoblot analyses of protein samples probed with antibodies against Rpn7 and 20S-α (A_2) after exposing middle aged flies for 7 days to the shown concentrations of CFZ or BTZ. (B) Relative (%) CT-L activity (B_1) and immunoblot analyses (B_2) of protein samples isolated from aged flies treated with CFZ or BTZ as in (A). GAPDH probing was used as loading reference. Comparisons were *vs*. control samples from flies grown in inhibitor-free culture medium. Bars, ± SD (n ≥ 2). *P < 0.05; **P < 0.01.

Suppl. Figure S4. Partial loss-of proteasome activity results in severe disruption of mitochondrial function and structure. (A) Relative mitochondrial ST3/ST4, FCCP/ST4 and ADP/ST3 ratios or actual ST2 values in isolated mitochondria from adult flies somatic tissues after the addition of 25 or 50 nM BTZ. (B) Oxygen consumption (Seahorse apparatus) in larvae tissues exposed (or not) to 1.5 μM BTZ (arrow); the time point of rotenone and antimycin addition is indicated by a dashed arrow. (C) High (upper panels) or low (lower panels) magnification EM micrographs showing the fine structure of mitochondria in muscles of young flies after treatment for 2 or 7 days with CFZ or BTZ; stars and arrows indicate disrupted mitochondrial cristae and membranes, respectively. Bars, ± SD; *P < 0.05; **P < 0.01.

Suppl. Figure S5. CFZ did not induce significant toxic effects during flies' developmental (oogenesis, embryogenesis) stages. (A) Comparative analyses of CFZ and BTZ effects in development following egg-laying, embryogenesis and completion of larval stages developmental stages in culture medium containing the shown concentrations of CFZ or BTZ. Relative pupae formation in comparison to non-treated controls [set to 100% (1)], was 102.13% ± 4.05 in 50 μM CFZ (normal flies eclosion), 91.845% ± 1.19 in 75 μM CFZ (normal flies eclosion), 80.73% ± 3.49 at 1 μM BTZ [rare or no pupae eclosion] and 46.215% ± 7.049 in 5 μM BTZ (increased larva lethality / no flies eclosion). (B) Longevity curves of adult flies (cultured in inhibitor-free medium) being treated (or not) during developmental stages with 50 μM CFZ. Flies' median lifespan and comparative statistics of the longevity assays are reported in Table S1. Bars, ± SD (n = 2). $^*P < 0.05$; $^{**}P < 0.01$.

Suppl. Figure S6. Epoxomicin (EPOX), BTZ and CFZ effects on proteasomal peptidases activity after being directly added in flies' tissue lysate containing intact proteasomes. Relative (%) CT-L (LLVY/β5), C-L (LLE/β1) and T-L (LRR/β2) proteasome activities in *Drosophila* somatic tissues lysate following the addition of the shown EPOX, BTZ and CFZ concentrations. Comparisons were *vs*. basal proteasomal activities in the absence of the inhibitor. Bars, ± SD (n = 3). $^*P < 0.05$; $^{**}P < 0.01$.

Suppl. Figure S7. Schematic representation of the experimental design used to assay the effects of CFZ and BTZ treatment in flies' tissues proteasomal peptidases activity. Flies were treated with culture medium containing (or not) 50 μM or 1 μM BTZ as indicated; arrows denote the time points where proteasomal peptidase activities were measured. More specifically in (A), flies were continuously exposed to the shown inhibitors and were transferred to fresh culture medium (containing –or not– the drug) every 4 days. A-F denotes time points of proteasome activities measurement. *A*: Exposure for 1 day to the PI; *B*: exposure for 4 days [in the culture medium of (A)] to the PI; *C*: exposure for 5 days to the PI (1 day in fresh culture medium containing the PI); *D*: exposure for 8 days [4 days in the culture medium of (C)] to the PI; *E*: exposure for 9 days to the PI (1 day in fresh culture medium containing the PI); *F*: exposed for 12 days [4 days in the culture medium of (E)] to the PI. In (B), flies were exposed for a period of 72 hrs to each drug and were then transferred to inhibitor-free culture medium; A-E denotes the time points of proteasome activities measurement. *A*: Exposure for 3 days to the shown PI; *B*: flies from (A) exposed for 1 day to inhibitor-free culture medium; *C*: flies from (A) exposed for 2 days to inhibitor-free culture medium; *D*: flies from (A) exposed for 3 days to inhibitor-free culture medium; and *E*: flies from (A) exposed for 4 days to inhibitor-free culture medium.

Suppl. Figure S8. NC001 effects on proteasome peptidases activity after being directly added in flies' tissue lysate containing intact proteasomes. Relative (%) CT-L (LLVY/β5), C-L (LLE/β1) and T-L (LRR/β2) proteasome activities in *Drosophila* somatic tissues lysate following addition of the shown NC001 (the chemical structure of the inhibitor is shown in the upper panel) doses. Comparisons were *vs*. control samples from flies grown in inhibitor-free culture medium. Bars, ± SD (n = 3). $^*P < 0.05$; $^{**}P < 0.01$.

Suppl. Figure S9. The β1-selective inhibitor NC001 was mildly toxic and enhanced the CFZ-mediated reduction in flies' longevity. (A) Relative (%) CT-L (LLVY/β5), C-L (LLE/β1) and T-L (LRR/β2) proteasome activities in flies' somatic tissues following treatment with the indicated doses of NC001 for 8 days. (B) Relative (%) ROS levels in the somatic tissues of young flies exposed to the shown doses of NC001 as in (A). (C) Representative immunoblot analyses of Rpn7, 20S-α and total ubiquitin (Ub) in lysates from somatic tissues of young flies treated with NC001 as in (A). (D, E) Longevity curves of flies continuously exposed (or not) to the indicated concentrations of NC001 (D) or to CFZ and NC001 (E); flies median lifespan and comparative statistics are reported in Supplementary Table S1. GAPDH probing (C) was used as reference. Comparisons were *vs*. control samples of inhibitor-free culture medium. Bars, ± SD (n ≥ 2). $^*P < 0.05$; $^{**}P < 0.01$.

Suppl. Figure S10. Our summarised findings in the fly model provide a reasonable explanation for the differential adverse effects of CFZ and BTZ in the clinic. We report that despite comparable cardiotoxicity, CFZ (as compared to BTZ) induces milder degenerative effects in the *Drosophila*

experimental model. The reduced toxicity of CFZ was due to increased (*vs*. BTZ) selectivity for the rate limiting for protein breakdown CT-L proteasomal activity.

Suppl. Figure S11. Calibration model for BTZ (A) and CFZ (B) in UPLC-MS studies. Error bars represent STDEV on a triplicate of analysis.

Supplementary Videos

Suppl. Video S1. Heart beats (~30 sec; representative video) of control flies.

Suppl. Video S2. Heart beats (~30 sec; representative video) of flies treated for 14 days with 75 μM CFZ.

Suppl. Video S3. Heart beats (~30 sec; representative video) of flies treated for 14 days with 5 μM BTZ.

Full blots of all the immunoblotting assays presented are shown at the end of the Supplementary Information Section.

Supplementary Methods

Proteasome inhibitors

The β1-selective proteasome inhibitor (NC001) was aliquoted and stored at -20°C after being diluted in DMSO; daily used aliquots were stored at 4°C. For the *in vivo* assays the inhibitor was directly added in flies' cultured medium. The DMSO concentration in flies' culture medium was always in between 0.05 to 0.1%. The synthesis of NC-001 (also known as Rub 1024) has been described previously[34].

Gustatory assay

Gustatory assays were done according to the method of Bahadorani and Hilliker (2008). Briefly, young flies were starved (or not) for 20 hrs on water-soaked Whatman paper and were then transferred for 2 hrs to vials containing standard culture medium mixed with 0.2% sulforhodamine B sodium salt (Acid-Red) (control) or culture medium containing the inhibitor under study and 0.2% sulforhodamine B sodium salt. The degree of abdomen redness for each fly was scored from grade 0 (colourless abdomen) to grade 5 (fully red abdomen).

Treatment with proteasome inhibitors during flies' developmental stages

To study the effects of CFZ and BTZ during developmental stages (oogenesis, embryogenesis and larval/pupal stages) equal number of young flies were cultured in the presence of BTZ (1, 5 μM) or CFZ (50, 75 μM) for 3 days. Flies were then removed from culture vials and the subsequent developmental stages of laid eggs were allowed to occur in the presence of the inhibitors under study. Equal number of the eclosed flies were collected and cultured in inhibitor-free medium for longevity assays. Survival curves were analysed by the Kaplan-Meier procedure and log-rank test. Statistical analysis of each lifespan experiment is presented in Table S1.

RNA extraction and Quantitative Real-Time PCR (Q-RT-PCR) analyses

Total RNA was extracted from dissected somatic tissues of flies using RNAzol (Molecular Research Center, Inc, OH, USA) and converted to cDNA with the Maxima First Strand cDNA Synthesis Kit of Thermo scientific, Inc (#K1642). cDNA was then subjected to Q-Real time-PCR analysis using the SYBR Green/ROX qPCR Master Mix of Thermo Scientific, Inc (#K0223). Primers were designed using the primer-BLAST tool (http://www.ncbi.nlm.nih.gov/ tools/primer-blast/) and were the following:
rpn11-F: ACAACAAGTCACTGGAGGACG, *rpn11*-R: TGCTTGCCCACGTTCTTGAT; *α7*-F: ACCGACGAATTGGTGGAGAG, *α7*-R: ACCCATTTCGAAGCGGAAGT; *β1*-F: GCGACGCATCTCTACAACAC, *β1*-R: CGAGGAAATGAAGCTGGGAGT; *β2*-F: AGCCACCGACCACCACCAAGA, *β2*-R: CCACAACGCGCACCTCACGA; *β5*-F: GCCATCTACCATGCCACCTT, *β5*-R: TTACCCAGCCGTCCTCCTTA; *atg8*-F: ACGCCTTCGAGAAGCGTCGC, *atg8*-R: CCAAATCACCGATGCGCGCC; *atg6*-F:

GTTCCTGCTGCCCTACAAGA, *atg6*-R: TCCACTGCTCCTCCGAGTTA; *keap1*-F: GCGCTCGTCAGCCCATTTT, *keap1*-R: GGATGCGCATAATTCCTCTTCTT; *trxr1*-R: ATTTTGAGTGCATGTCGGCG, *trxr1*-F: GACACCAGTGGAACTACCCG; *hsp70*-F: AAGAACCTCAAGGGTGAGCG, *hsp70*-R: CGTCGATGGTCAGGATGGAG. The ribosomal gene rp49 (*rp49*-F: AGCACTTCATCCGCCACC, *rp49*-R: ATCTCGCCGCAGTAAACG) was used as a normalizer as described before[61].

Preparation of tissue protein extracts, immunoblot analysis and antibodies used

Isolated somatic tissues were homogenised on ice in NP-40 lysis buffer containing protease inhibitors and were analysed by SDS-PAGE and immunoblotting as described previously[59-61]. Primary and secondary antibodies were applied for 1 h at RT. The antibodies against Rpn7 (p42A) (sc-65750), 20S-α (sc-65755), Ubiquitin (sc-8017) and GAPDH (sc-25778) were from Santa Cruz Biotechnology Inc. The anti-AGEs antibody was from Cosmo Bio (KAL-KH001-01). The antibody against the *Drosophila* β5 proteasome subunit was a gift from Dr. Maria Figueiredo-Pereira (Dept of Biological Sciences, Hunter College of the City University of NY, USA).

Detection of protein carbonyl groups and measurement of reactive oxygen species (ROS)

For the detection of protein carbonyl groups the OxyBlot protein oxidation detection kit (Millipore, Billerica, MA; #s7150) was used as per manufacturer's instructions.

ROS were assayed as previously described[59]. The fluorescent dichloro-dihydro-fluorescein was measured using a VersaFluor Fluorometer System (Bio-Rad Laboratories, Hercules, CA, USA) (excitation 490 nm, emission 520 nm).

Measurement of proteasome and cathepsins B, L peptidases activity

For measuring proteasome peptidases activity in dissected flies' somatic tissues, ovaries or larvae, as well as in cells; isolated samples were lysed on ice by using buffers suitable for the isolation of either 26S (0.2% Nonidet P-40, 5 mM ATP, 10% glycerol, 20 mM KCl, 1 mM EDTA, 1 mM DDT, 20 mM Tris, pH 7.6) or 20S (0.5% Triton X-100, 250 mM NaCl, 3 mM EDTA, 3 mM EGTA, 20 mM Tris, pH 7.6) proteasomes. Lysates were cleared with centrifugation at 19,000 xg (4°C), and, after protein content adjustment with Bradford, supernatants were immediately used to determine proteasome proteolytic activities, as described previously[59]. The cathepsins B, L activity in flies' tissues was assayed as described before[61]. In either proteasome or cathepsin assays the hydrolysis of the fluorogenic peptides was recorded (excitation, 350 nm; emission, 440 nm) in a Versa Fluor™ fluorometer system.

Mitochondria isolation and measurement of mitochondrial respiration or O_2 consumption in larvae tissues

Mitochondria were isolated as described by Ferguson et al. (2005). Briefly, somatic tissues of flies were homogenised in ice-cold isolation buffer (0.32 M sucrose, 10 mM EDTA, 10 mM Tris/HCl, pH 7.3) containing 2% (w/v) BSA. Samples were filtered through a layer of gauze which was then washed with additional isolation buffer up to a final volume of 1.5 ml. Following centrifugation for 10 min at 2,200×g, the pellet was washed with BSA-free isolation buffer and resuspended in 200 µl of the same buffer. Protein content of isolated mitochondria was assayed by Bradford.

Mitochondrial respiration was determined using a Clark-type O_2 electrode connected to a computer-operated Oxygraph control unit (Hansatech Instruments, Norfolk, U.K.) as previously described (Cogliati et al., 2013). Freshly isolated mitochondria (150 µg of protein) were added to the respiration buffer (120 mM KCl, 5 mM KH_2PO_4, 3 mM Hepes, 1 mM EGTA, 1 mM $MgCl_2$, 0.2% BSA, pH 7.2) containing 5 mM glutamate/2.5 mM malate. Basal O_2 consumption was recorded (state 2) and after 2 min 500 µM ADP was added (*State 3*; indicates rate of ATP production, O_2 consumption), followed by 6 µM oligomycin (*State 4*; denotes coupling) and 100 nM of the uncoupler (causes maximal respiration) carbonyl cyanide p-trifluoromethoxyphenylhydrazone (FCCP) (*State FCCP*). In all experiments, the temperature was maintained at 25°C and the total reaction volume was 300 µl. The respiratory control ratio (PCR) was calculated as the ratio of State 3 to State 4 (*ST3/ST4*).

In vivo O_2 consumption measurements were made using the XF24 Extracellular flux analyzer (Seahorse Bioscience) (Da-Rè et al., 2014). Briefly, each dissected 3rd instar larvae (body wall preparations) was placed into a well of an islet capture 24-well microplate. Larva held in place with a

grid and covered with HL3A medium. The instrument was maintained at a temperature of 25°C. Basal O_2 consumption rates were measured three times and reported in picomoles/min. Twenty preparations per experiment were used.

Isolation of semi-intact hearts from adult flies

For visualizing the *Drosophila* beating heart, young w^{1118} flies were dissected as described before (Vogler and Ocorr, 2009) with minor modifications. The entire procedure was performed in an oxygenated, artificial haemolymph (AH) solution (108 mM Na^+, 5 mM K^+, 2 mM Ca^{2+}, 8 mM $MgCl_2$, 1 mM NaH_2PO_4, 4 mM $NaHCO_3$, 10 mM sucrose, 5 mM trehalose, 5 mM HEPES, pH 7.1) at room temperature. Briefly, flies were anesthetised with short term exposure to cold. The insects were then placed (dorsal side down) into a Petri dish coated with a thin layer of petroleum jelly to avoid any movement and dissected using spring scissors (Vannas Spring Scissors, straight/2.5mm). By using forceps and suitable capillaries (diameter smaller than 40 microns) the ventral abdominal cuticle and all internal organs were removed in order to clearly see the heart tube. Recordings of heart activity were acquired using a BMS (Breukhoven microscopy systems/3 MB) digital microscope camera mounted on a BMS microscope. Manual measurements (heart beats recording) were obtained for ~30 sec per sample; recorded movies (see, Supplementary Information) were ~30 sec in duration.

Sample preparation for Confocal Laser Scanning Microscopy (CLSM)

Young UAS $mito^{GFP}/Gal4^{MEF2}$ flies were dissected and cardiac tubes were isolated in AH solution. Isolated hearts were then fixed in 4% paraformaldehyde in PBS for 15 min and washed in PBS. For nuclei visualization samples were counterstained with DAPI (Molecular Probes, #D1306). The mitochondria in cardiomyocytes of *Drosophila* heart tubes sections were imaged using a Digital Eclipse C1 (Nikon, Melville, NY, USA) confocal microscope equipped with a Nikon Plan APO 60.0×/1.40 oil immersion objective. Z-stacks with a step size of 1 μm were taken using identical settings. Each stack consisted of 18 plane images.

Electron microscopy

Sample preparation and Electron microscopy (EM) viewing were done as described by Debattisti et al. (2014) with minor modifications. Briefly, muscles of adult flies were incubated for 2 hrs (at RT) in fixation solution containing 4% paraformaldehyde, 2% glutaraldehyde in 0.1 mM sodium cacodylate buffer (pH 7.2) and were then washed overnight at 4 °C. Samples were post-fixed with 1% OsO_4 and were then embedded in Epon. EM images were acquired from thin sections using a transmission electron microscope Tecnai 12 (FEI, North America NanoPort, Hillsboro, Oregon, USA).

Sample preparation and Ultra-High Performance Liquid Chromatography – Mass spectrometry (UPLC-MS)

For analyses, dissected flies' somatic tissues were treated for protein precipitation. Briefly, somatic tissues from 50 flies were placed into an eppendorf with 50 μl of cold MetOH and homogenized with a pestle. The volume of cold MetOH was slowly increased to 500 μl in order to attain a complete homogenization. Following a centrifugation step at 12,000 xg for 3 min (4ºC) the supernatant was collected and 10 μl of the isolated supernatant were analysed by UPLC-MS. For analysis of flies' culture medium, a blank and samples containing the indicated concentration of either CFZ or BTZ were used; both samples were extracted with MetOH.

Samples were analysed in an Advance™ UPLC system coupled to an EVOQ™ Elite Triple Quadrupole Mass Spectrometer (Bruker). Separation was carried out on a Acquity® HSS T3 (Waters) column (100 mm x 2.1 mm, 1.8 μm) heated at 40°C with a gradient of water containing 0.1% (v/v) formic acid (A) and acetonitrile (B) at a flow rate of 0.4 mL/min. Injection volume was of 5 μL. Elution started at 20% B and reached 60% B in 5 minutes; then was increased to 100% B in 0.5 min. These conditions were maintained for 1.5 minutes before going back to initial conditions for 2 min. Ionization was achieved with a heated ESI source operated in both modes (negative and positive). A tension of 4.5 kV was applied to the capillary both in ESI(+) and ESI(-). Heated probe and cone temperatures were both set at 300°C whereas probe and cone desolvatation gas (nitrogen) flows were set at 40 and 20 units, respectively. Nebulizer gas flow was set at 50 units and exhaust gas was on. Argon under a vacuum of 1.5 mTorr was used as collision gas. In order to increase specificity in the measurements, acquisition was performed using multiple reactions monitoring (MRM). Positive mode

MRM functions were used for BTZ due to the better ionization. More specifically, the transitions 383.2>364.9 and 383.2>321.9 were chosen for BTZ as quantification and confirmation transitions, respectively, and monitored from 0.3 to 4 min. Collision energies were set at 8 V and 15 V. Negative mode MRM functions were monitored from 4 to 7 min and CFZ was detected using 720.5>289 as the quantification transition, and 720.5>261 as the confirmation transition; associated collision energies were of 34 V and 40 V, respectively. 2-hydroxycinnamic acid was used as internal standard (IS) for optimum quantification and was detected using the single ion monitoring (SIM) 163.1>119.10 with a collision energy of 10 V.

Analysis of tissue samples

A first step for the quantitation of analytes was the establishment of standard calibration curves and the semi-validation of the method. Thus, standard solutions of BTZ in six concentration levels ranging from 1 to 33 ng/mL were analysed in triplicates and IS at a concentration of 50 ng/mL. A calibration curve as a function of analyte/internal standard areas versus the ratio of their concentrations was built (Supplementary Fig. S11A). For CFZ, analytical triplicates of standard solutions in nine concentration levels ranging from 0.6 to 100 ng/mL were used to build the calibration curve (Supplementary Fig. S11B). Linearity was then evaluated by coefficients of determination (R^2) from the linear regression using the least squares approach. $R^2>0.995$ was observed for both targeted compounds and plots of residuals plots of residuals displayed homogenous distributions. All samples used for the calibration model were analysed in triplicates and were injected randomly throughout the sequence.

In order to monitor any eventual cross contaminations or carryover effects, blanks were analysed every 10 injections. A QC sample that consisted of both analytes at 15 ng/mL and IS was analysed in triplicate at the beginning and at the end of the sequence. Recovery experiments were performed through measurement of spiked samples with known amounts of standards. Specifically, 2 aliquots of control samples (tissues from flies not treated with BTZ or CFZ) were spiked with analytical standards of BTZ and CFZ and then were supplemented with IS solution and MeOH. Final concentration in these samples was of 10 ng/mL for BTZ and CFZ, and 30 ng/mL for the IS.

Recovery experiments were also performed. Known amounts of analytes were added in 2 aliquots of a control sample. Recovery of the investigated compounds in spiked matrix was of 86% for BTZ and of 91% for CFZ with RSD of 14% and 9%, respectively (n=6). None of the targeted compounds could be detected in the blanks and RSD of Area ratio (Analyte/IS) of the QC samples (n=6) were 8% for BTZ and 4% for CFZ. Limit of Quantification (LOQ) were determined by injecting a series of dilute solutions of the standards until the signal-to-noise ratio 10:1. In the conditions of the analysis and taking into account all dilution factors, LOQ was determined at 2 ng/mL for BTZ and of 1 ng/mL for CFZ. For the measurements of the working samples, 50 μL of each were transferred to a vial into which 50 μL of IS solution were added (50 μL in MeOH) resulting to 30 ng/mL per sample.

Statistical analysis

Shown experiments were performed at least in duplicates and data points correspond to the mean of the independent experiments. For statistical analyses, the MS Excel and the Statistical Package for Social Sciences (IBM SPSS; version 19.0 for Windows, NY, USA) were used. Statistical significance was evaluated using one-way analysis of variance (ANOVA). Error bars indicate standard deviation (SD); significance at $P<0.05$ or $P<0.01$ is indicated in graphs by one or two asterisks, respectively.

Supplementary References

Bahadorani, S. & Hilliker, AJ. Cocoa confers life span extension in *Drosophila melanogaster*. *Nutrition Research* **28,** 377-82 (2008).

Cogliati S. et al. Mitochondrial cristae shape determines respiratory chain supercomplexes assembly and respiratory efficiency. *Cell* **155**, 160–171 (2013).

Da-Ré C. et al. UCP4C mediates uncoupled respiration in larvae of *Drosophila melanogaster*. *EMBO Rep*. **15**, 586-91 (2014).

Debattisti, V., Pendin, D., Ziviani, E., Daga, A. & Scorrano, L. Reduction of endoplasmic reticulum stress attenuates the defects caused by *Drosophila* mitofusin depletion. *J Cell Biol* **204**, 303–312 (2014).

Ferguson, M., Mockett, M.J., Shen, Y., Orr, W.C. & Sohal, R.S. Age-associated decline in mitochondrial respiration and electron transport in *Drosophila melanogaster*. *Biochem J* **390**, 501–511 (2005).

Vogler, G. & Ocorr, K. Visualizing the beating heart in *Drosophila*. *J. Vis. Exp*. **31,** pii: 1425 (2009).

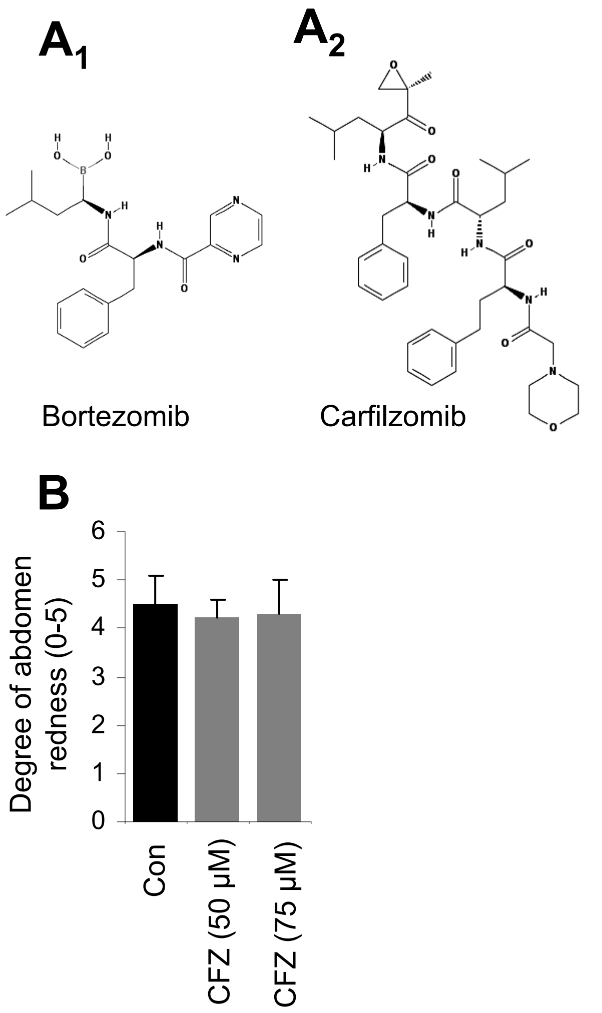

Tsakiri et al. Suppl. Fig. S1

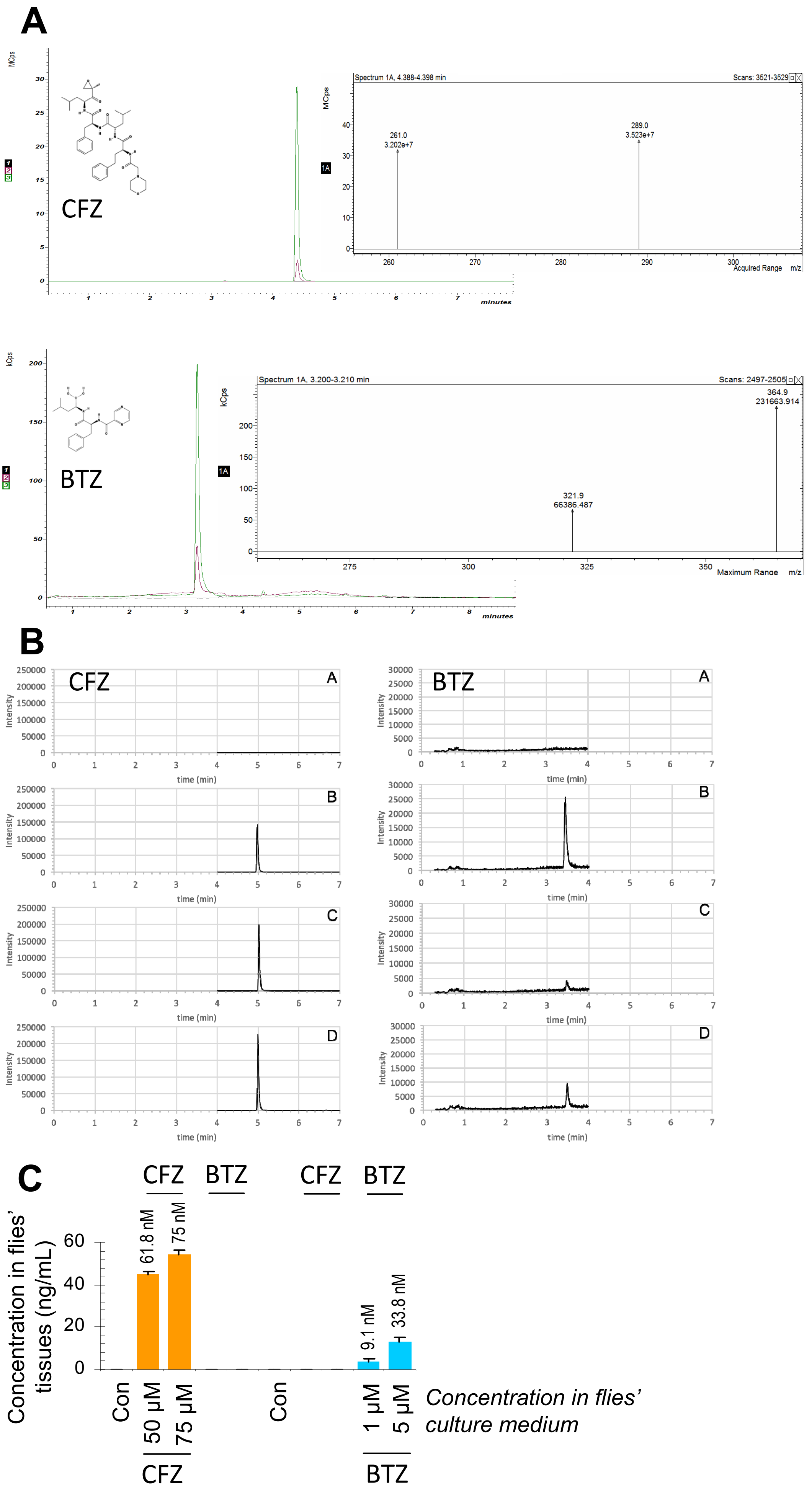

Tsakiri et al. Suppl. Fig. S2

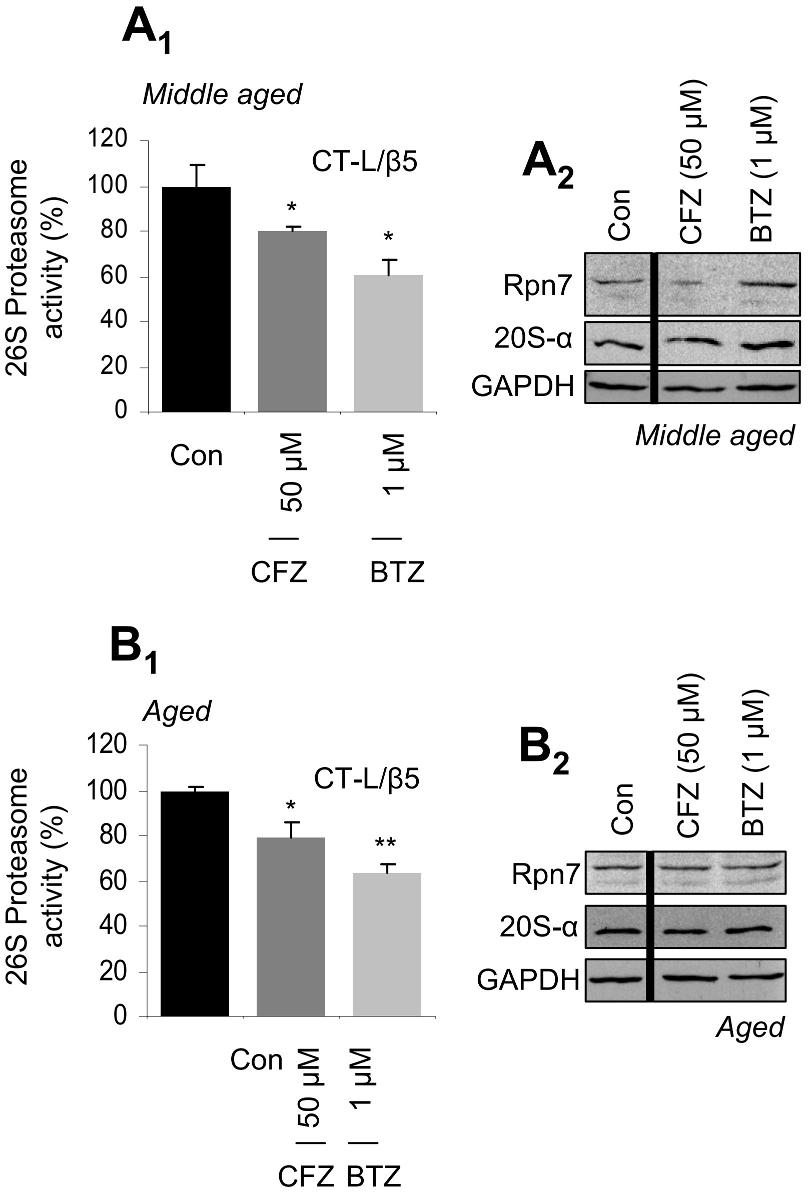

Tsakiri et al. Suppl. Fig. S3

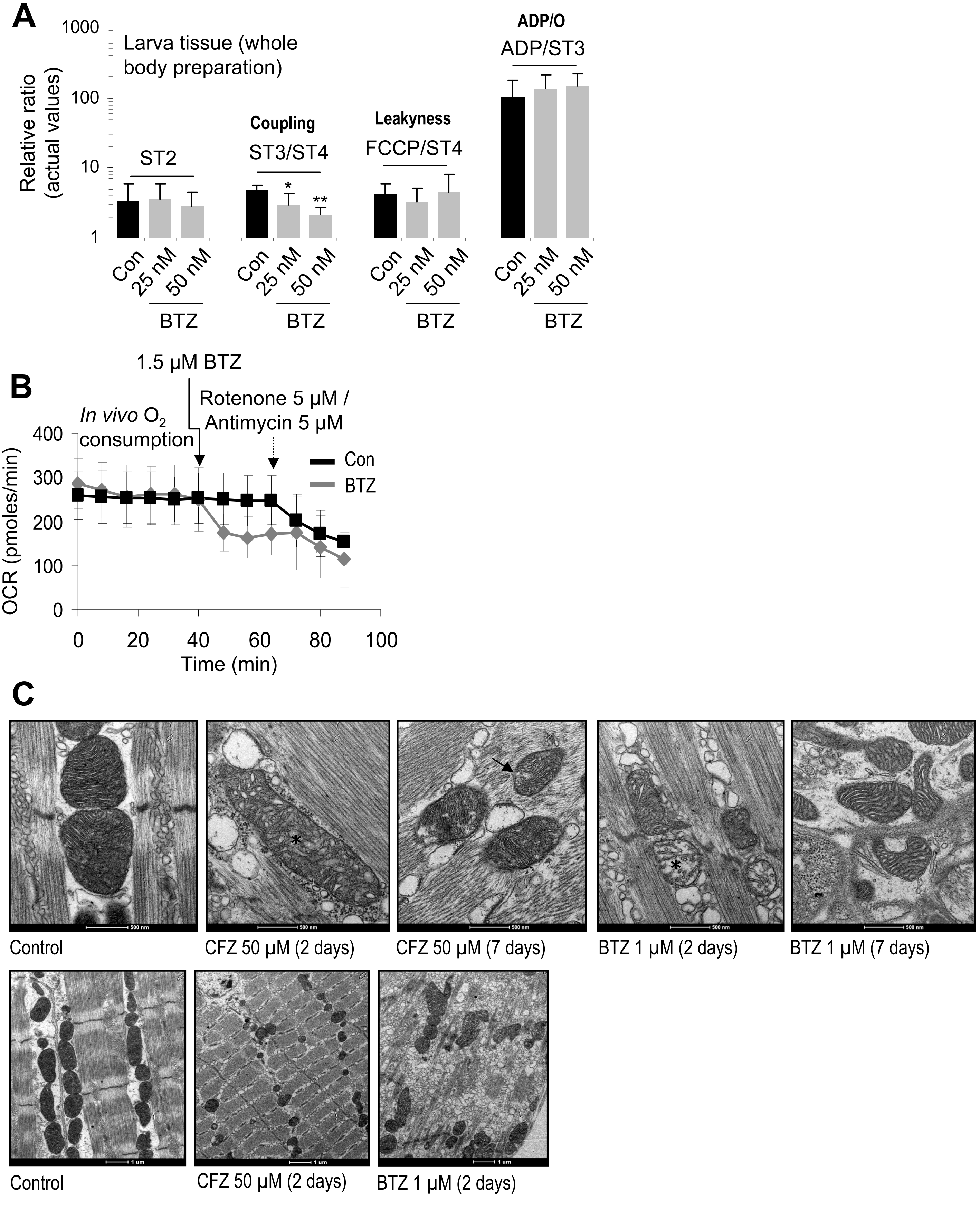

Tsakiri et al. Suppl. Fig. S4

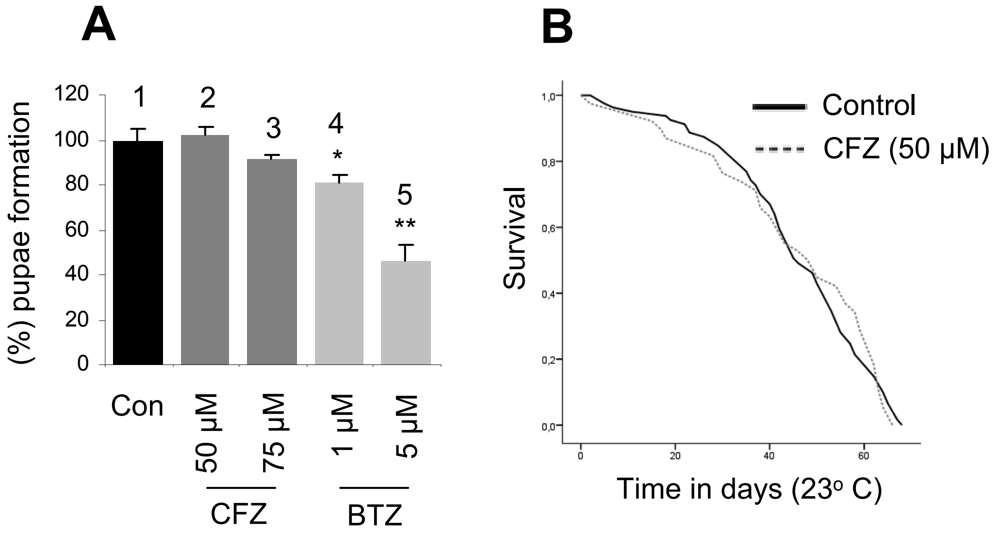

Effects to flies eclosion

1: Normal flies eclosion
2, 3: Normal flies eclosion
4: Rare (or no) flies eclosion
5: Increased larva lethality / no flies eclosion

Tsakiri et al. Suppl. Fig. S5

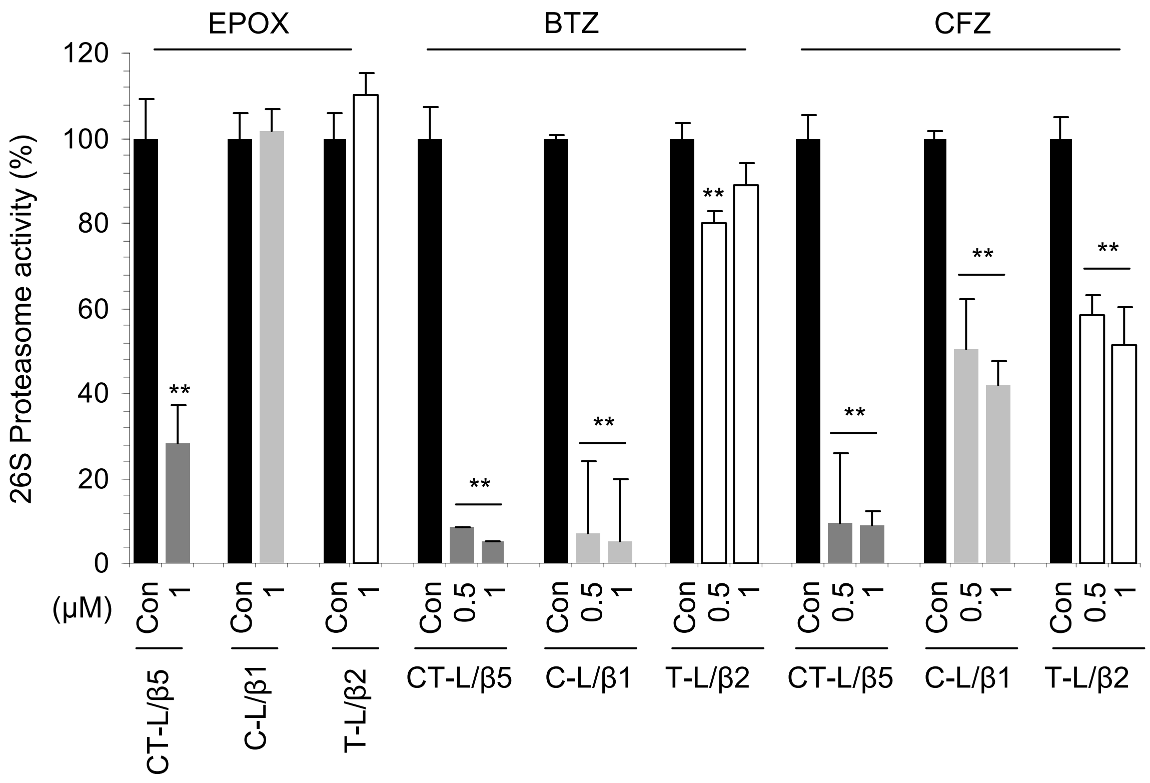

Tsakiri et al. Suppl. Fig. S6

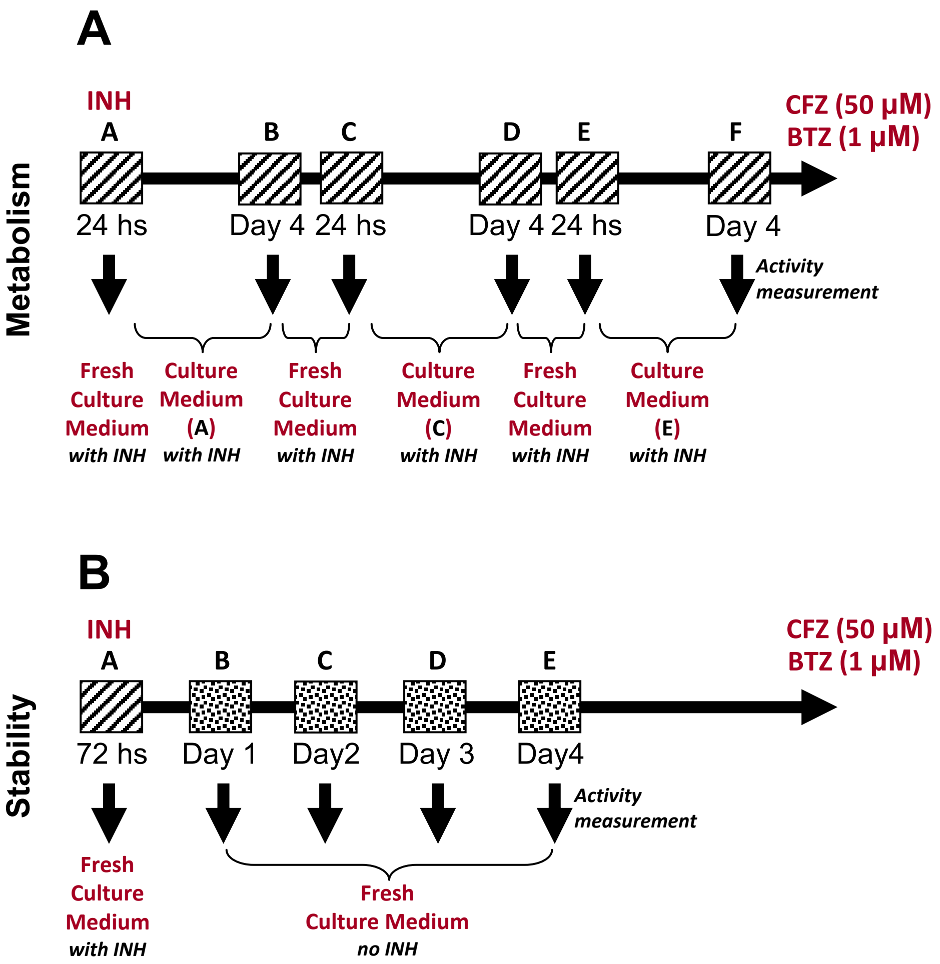

Tsakiri et al. Suppl. Fig. S7

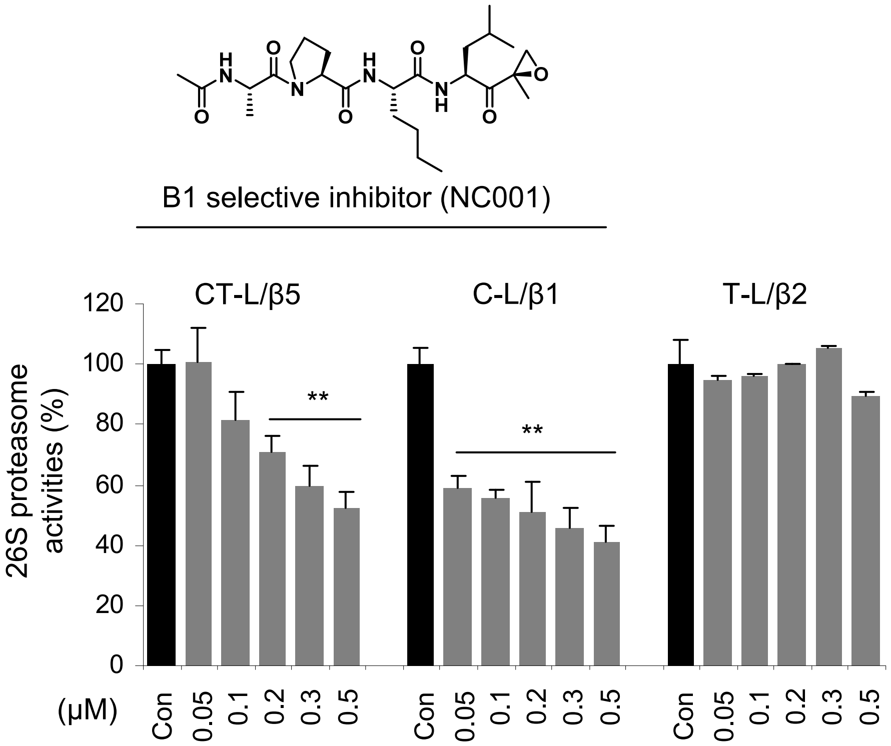

Tsakiri et al. Suppl. Fig. S8

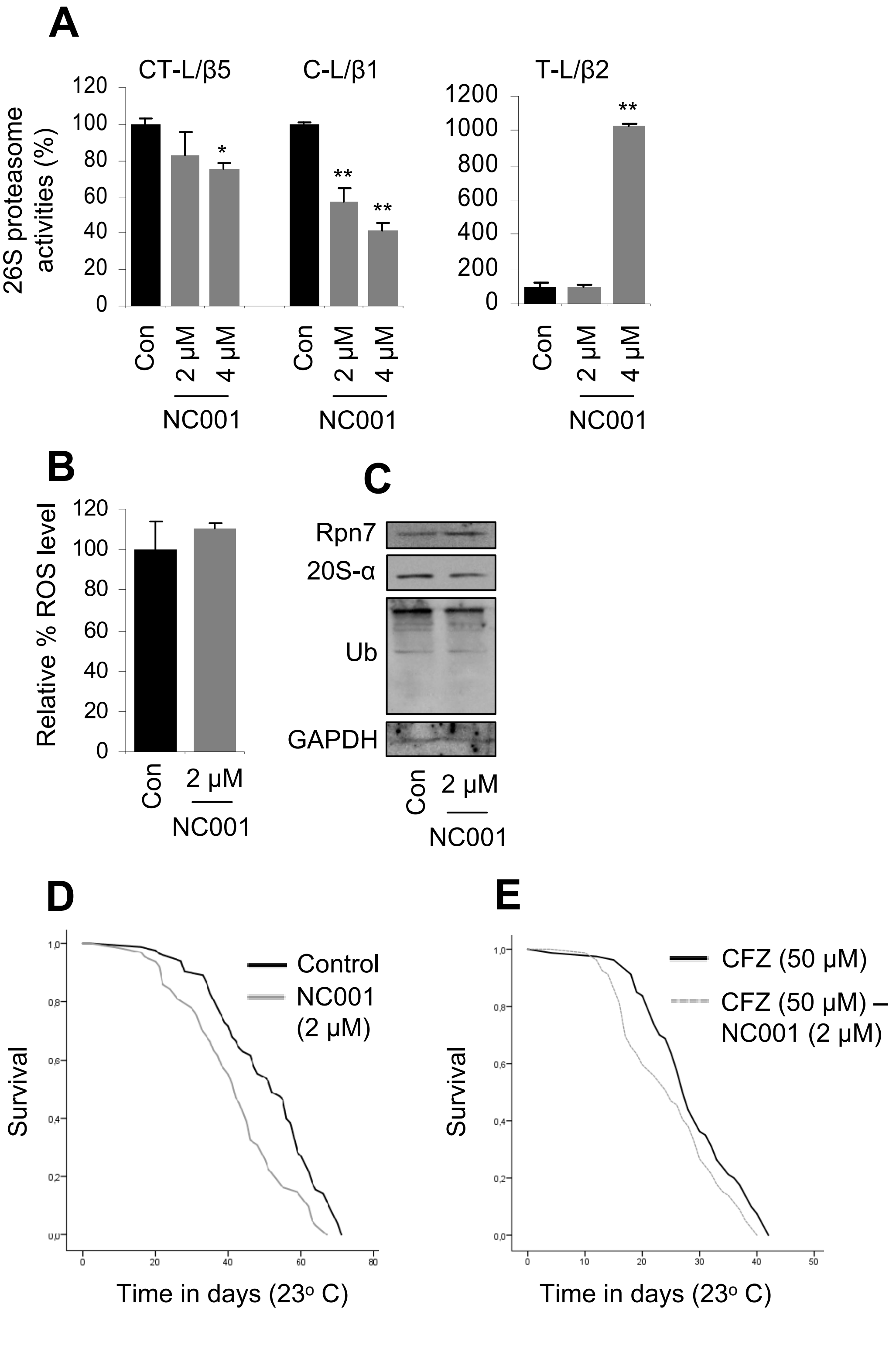

Tsakiri et al. Suppl. Fig. S9

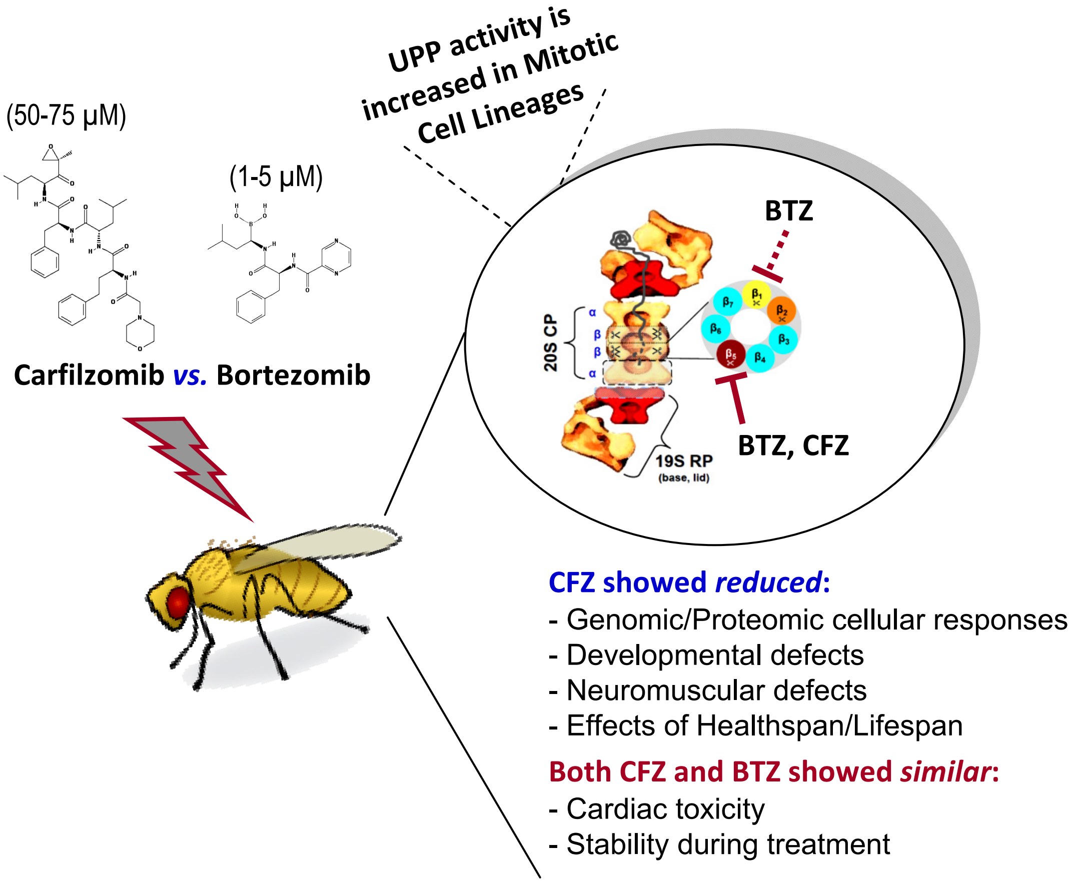

Tsakiri et al. Suppl. Fig. S10

A

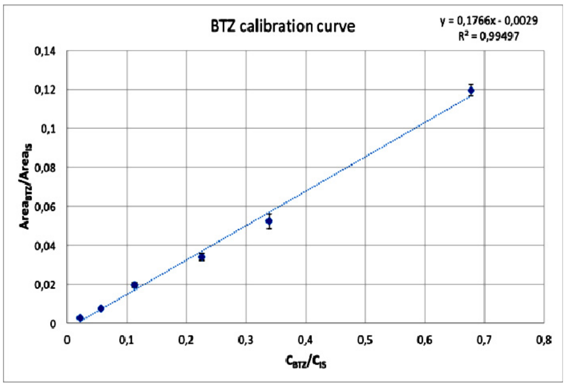

B

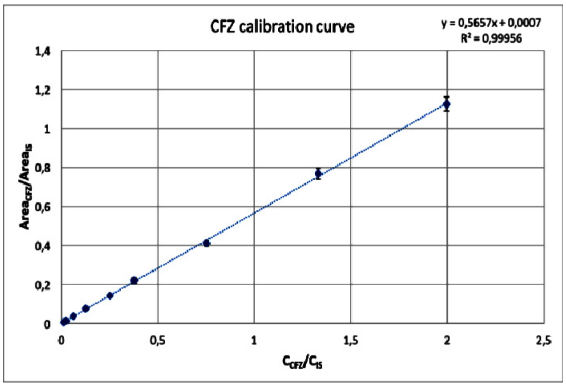

Tsakiri et al. Suppl. Fig. S11

Supplementary Table S1. Summary of lifespan experiments.

Figure	Sample	Mean Lifespan (LF) +/- s.e.m. (Days)		Median LF +/- s.e.m. (Days)		% Median LF vs. control	Max (Days)	Log Rank P Value			
								Control	50 µM CFZ	1 µM BTZ	*Total animals died/Total*
Fig. 3D	Control	44.893	1.115	45	1.088	100	69		0.000	0.000	123/147
	50 µM CFZ	28.126	0.863	27	1.880	61.36	46	0.000		0.000	123/144
	1 µM BTZ	19.802	0.471	21	0.236	46.66	27	0.000	0.000		88/99
								Control	Embryoogenesis - 50 µM CFZ	*Total animals died/Total*	
Fig. S5B	Control	45.470	1.818	46	3.037	100	69		0.791	67/80	
	Embryoogenesis - 50 µM CFZ	45.480	1.961	49	3.804	106.52	66	0.791		76/80	
								Control	2 µM NC001	*Total animals died/Total*	
Fig. S9D	Control	50.167	1.556	52	2.903	100	71		0.000	79/85	
	2 µM NC001	41.382	1.772	42	1.956	80.76	67	0.000		62/68	
								50 µM CFZ	50 µM CFZ - 2 µM NC001	*Total animals died/Total*	
Fig. S9E	50 µM CFZ	28.238	0.887	27	0.813	100	42		0.009	80/80	
	50 µM CFZ - 2 µM NC001	24.787	0.934	25	1.845	92.59	40	0.009		79/80	

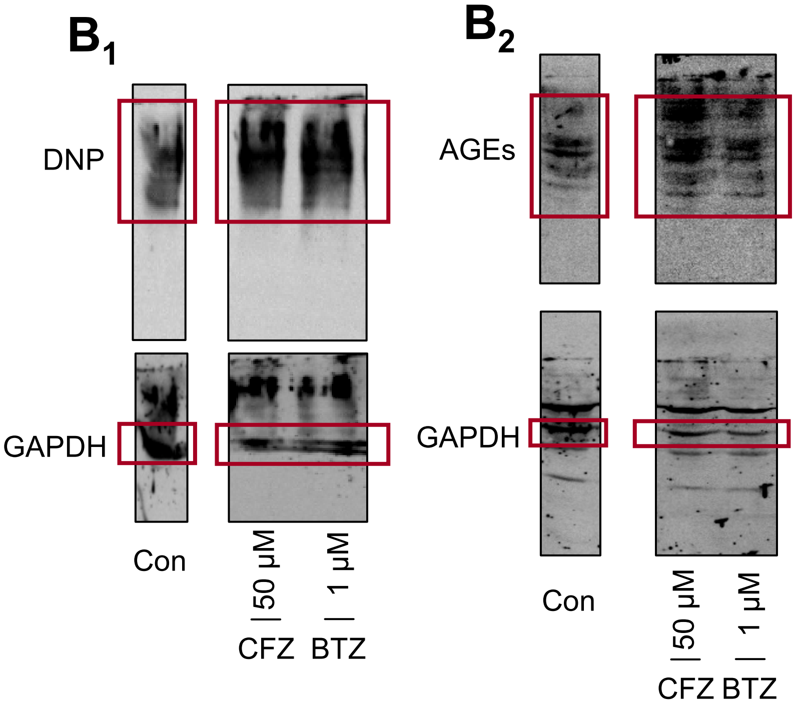

Tsakiri et al. Figure 1

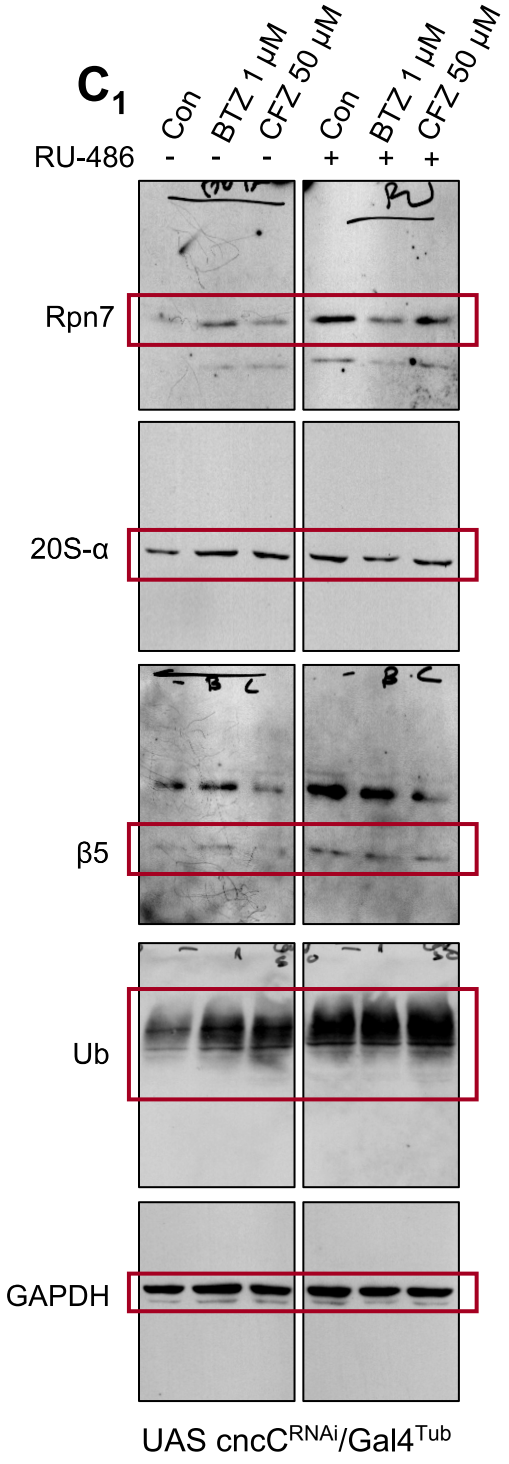

Tsakiri et al. Figure 2

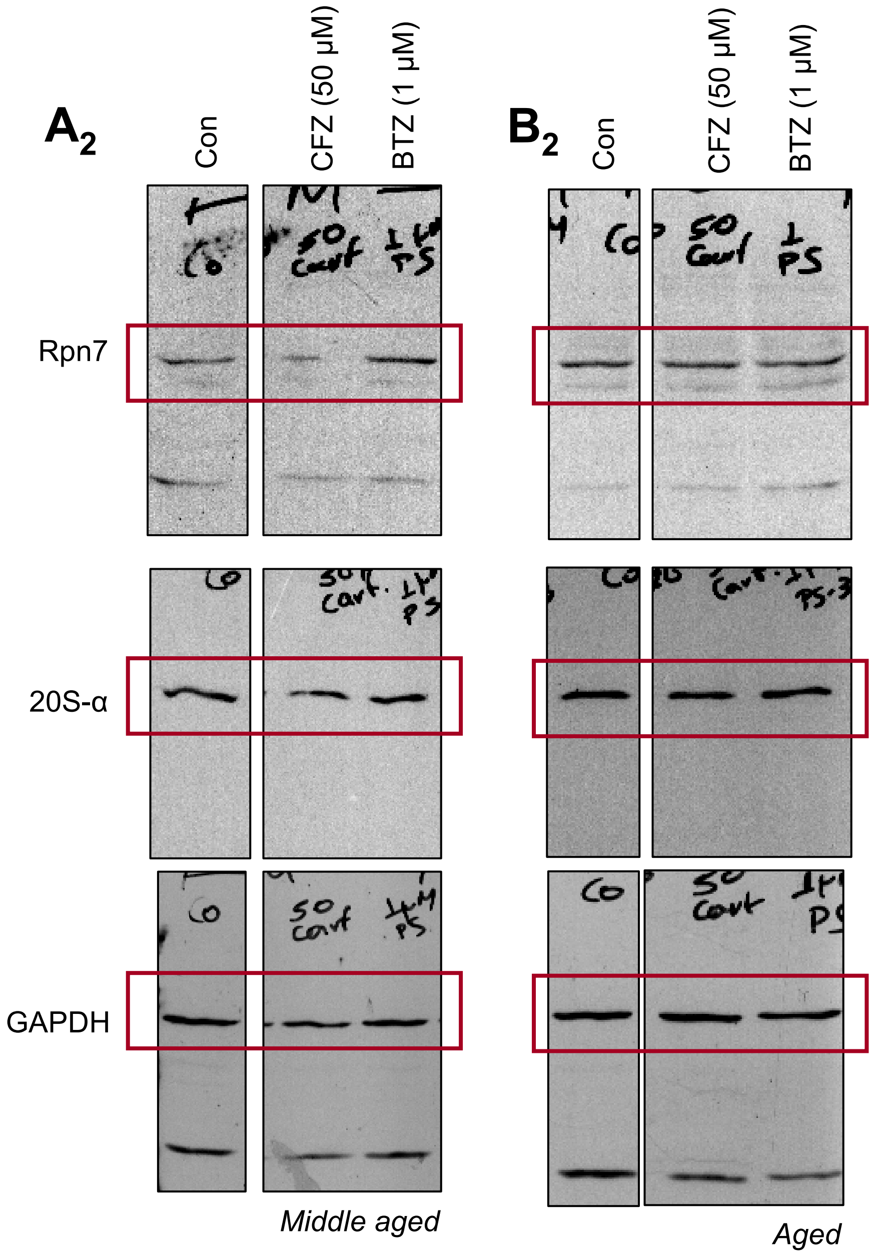

Tsakiri et al. Suppl. Fig. S3

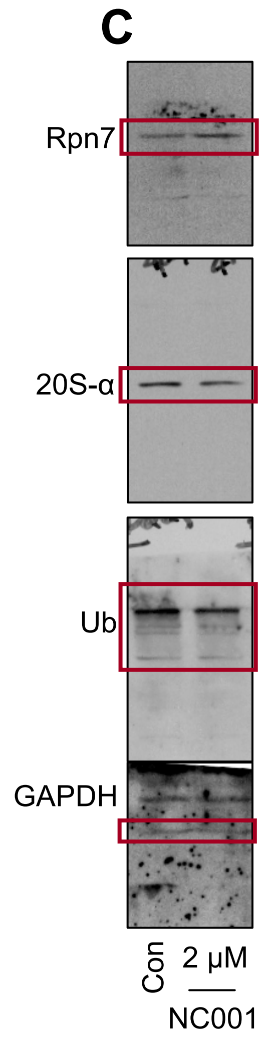

Tsakiri et al. Suppl. Fig. S9
